# Human health risks of potentially toxic elements in the soil-crop system of a coal mining area, Moatize, Mozambique

**DOI:** 10.1007/s10661-026-15147-x

**Published:** 2026-04-10

**Authors:** Micaela Arlete José Chapo Cossa, Hassina Mouri, Robert B. Finkelman, Vicente Albino Manjate, Kim Dowling

**Affiliations:** 1 Department of Geology, Faculty of Science, University of Johannesburg, Johannesburg, 2006 South Africa; 2 University of Texas at Dallas, Richardson, TX 75080 USA; 3 Ministry of Mineral Resources and Energy, National Institute of Mines, Maputo, Mozambique; 4 School of Science, STEM College, RMIT University, Melbourne, VIC 3001 Australia

**Keywords:** Bioaccumulation Factor, Cowpea, Geo-accumulation Index, Toxic risk index, Zea mays

## Abstract

This study investigates the pathways by which potentially toxic elements (PTEs) enter the environment during coal mining activities in Moatize, Mozambique. Using Inductively Coupled Plasma Mass Spectrometry (ICP-MS) and Ion Chromatography (IC), samples of coal (n = 11), soil (n = 18), sediment (n = 4), and food crops (n = 15) were analysed. Results confirm significant accumulation of PTEs in coal. Environmental contamination is evident, as mean concentrations of Al, Co, Fe, and Mn in soil and sediments surpass the standards established by the Food and Agriculture Organization/World Health Organization (FAO/WHO). In crops, Cr (0.92–4.16 mg/kg) and Ni (0.47–1.18 mg/kg) exceeded the permissible limit (0.1 mg/kg) in 60% of samples. Enrichment factors (EF > 2) for As, Co, and Ni indicate an attribution to anthropogenic activity, whereas an extreme ecological risk (Er > 320) for As and Co suggests that mining activities are the predominant sources. The Toxicity Risk Index (TRI) indicates higher ecological stress in soils (65% high TRI) than sediments, primarily due to As and Cr toxicity. Human health assessments confirmed non-carcinogenic risks, with Hazard quotients (HQ > 1.0) for Al, Mn, and Fe in adults and Al and Fe in children. Bioaccumulation factors were higher in Cowpea than *Zea mays*, posing a greater risk to consumers of local food crops. Although the cumulative hazard index (HI) for Cr, Cu, Ni, and Zn remained low, Cr posed the highest carcinogenic risk, especially for adults. These findings emphasise the importance of regular monitoring and sustainable land-use practices to reduce ecological and human health risks and safeguard the Moatize ecosystem.

**Supplementary Information:**

The online version contains supplementary material available at 10.1007/s10661-026-15147-x.

## Introduction

Coal, an organic-rich, combustible sedimentary rock, is mainly composed of carbon but can contain up to 50% mineral matter (Finkelman et al., 2019). It also contains significant amounts of hydrogen, oxygen, and sulfur, along with moisture and trace elements (Kumar et al., 2022). As the most abundant fossil fuel (Niu et al., 2017), it remains the primary energy source, accounting for over 41.1% of global electricity production (Kookhaei et al., 2025). Coal mining provides a substantial boost to national economies and will remain important for decades, benefiting both developing and developed countries (Chugh et al., 2023). However, mining operations release harmful substances, alter land, and pollute air, water, and soil, impacting biodiversity and human health (Finkelman et al., 2021; Yiika et al., 2023). Additionally, coal mining contributes to climate change through greenhouse gas emissions, making up 39% of global CO2 emissions (Gopinathan et al., 2023).

The use of sediment and soil geochemical signatures is crucial for determining provenance, depositional environment, redox conditions, and contamination by PTEs (Afahnwie et al., 2025a; Djibril et al., 2024; Jean-Lavenir et al., 2025; Yiika et al., 2025). Soil, a vital natural resource for sustainable development and biomass production, is heavily impacted by industrialisation and coal mining, especially near mining sites (Sigue et al., 2025). PTEs in soil originate from both natural sources and increasingly from human activities such as urban development, industrialisation, and mining (Jean-Lavenir et al., 2024; Suh et al., 2025). PTEs are pervasive environmental pollutants that have attracted increasing attention in recent decades due to their persistence, non-biodegradability, toxicity, and propensity to bioaccumulate in soil and humans. (Kachoueiyan et al., 2024a; Tiabou et al., 2024a; Yiika et al., 2022). Such elements are found in soil and sediments, with their form and mobility influenced by physicochemical factors such as organic matter content, pH, conductivity, and salinity (Kachoueiyan et al., 2023; Taghavi et al., 2025). Coal mining stands as a major source of PTEs, dispersing various elements into the environment (Bai et al., 2023). Excess accumulation of these persistent, non-degradable PTEs irreversibly reduces soil fertility, crop yields, and quality (Tiabou et al., 2024b; Yiika et al., 2024). Though soil can act as a sink for PTEs (Sanad et al., 2025a; Tiabou et al., 2024c), their leaching, especially of Cd, Co, Mn, Ni, S, and Zn (Ribeiro & Flores, 2021), poses a serious contamination risk (Rouhani et al., 2023a). Elevated PTE levels harm ecosystems, decrease microbial diversity, damage plants, and hamper agricultural productivity (Afahnwie et al., 2025b; Rashid et al., 2023). As human activities increase, so do environmental PTE levels. These elements enter the food chain, creating health risks for humans through exposure routes such as dermal contact, ingestion, and inhalation (Afahnwie et al., 2025c; Babaniyi et al., 2024; Sanad et al., 2025b). Crops absorb and accumulate PTEs, which can pass to humans through daily food intake (Tiabou et al., 2025a). This leads to significant health concerns, including skin irritation, respiratory issues like black lung disease, potential cancer, neurological damage, gastrointestinal problems, and developmental disorders (Budi et al., 2024; Suh et al., 2025; Yiika et al., 2024).

Globally, numerous studies consistently show that food crops grown in contaminated soil accumulate elevated levels of PTEs. This widespread problem is well-documented across various regions, including Africa (Ogbuene et al., 2024; Oladeji et al., 2024; Tiabou et al., 2025b), Europe (Akkoca et al., 2024; Alekseenko et al., 2025), Asia (Chakraborty et al., 2023; Raj & Das, 2025), and America (Romero-Crespo et al., 2023; Greenberg & Schneider, 2025).

In the Moatize district of Mozambique, the main environmental and public health threats come from coal mining activities and related community practices (Mahumane, 2025). These issues spread through several coal pathways: (1) chemical leaching caused by acid mine drainage (AMD), where sulfide minerals like pyrite in coal waste oxidase and produce sulfuric acid, which releases toxic elements like Al, Fe, and Mn into groundwater and nearby soils; (2) the spread of coal dust through the air during large-scale open-pit mining and material transport, which deposits fine particles onto distant agricultural fields; and (3) water runoff from coal stockpiles and uncontained reservoirs washing into local floodplains during the rainy season. Additionally, the unregulated domestic use and disposal of coal ash from brick kilns introduces concentrated toxic elements directly into the topsoil of residential and agricultural areas, altering soil chemistry and increasing the availability of toxic elements such as Cr and Ni. These pathways facilitate the entry of PTEs into the food chain via staple crops, where they are absorbed and may accumulate over time (Ullah et al., 2025). Once ingested, these elements can accumulate in the human body, potentially leading to serious health problems, including cancer, neurological damage, and kidney disease (Hossain et al., 2025).

Despite these known risks, a significant data gap exists regarding PTEs contamination in Moatize, particularly concerning soils and food crops. While some studies (de Oliveira et al., 2024; Weiler et al., 2020) are available, only one study by Marove et al. (2020) explicitly examined the leaching of hazardous elements from coal and coal ash. Another study (Marove et al., 2022) found that river soils and sediments were highly polluted, with a pollution load index (PLI) ranging from 1.11 to 1.85. As a result, this study represents the first comprehensive investigation to analyse a wide range of elements, including Al, Cr, Cu, Fe, Mn, Mo, Ni, S, Si, V, and Zn across the entire Moatize region, filling a critical data gap. The research aims to: (i) evaluate PTE concentrations in soils, sediments, and staple crops; (ii) determine soil-to-plant transfer factors for each PTE; (iii) assess local human health risks from contaminated crop consumption; and (iv) map the spatial distribution of elevated PTEs to understand regional variability and environmental impact. The framework uses analytical and statistical methods to develop a quantitative risk assessment model for soil and crop contaminants, applicable to coal mining regions worldwide. Contamination maps will highlight high-risk areas, guiding future remediation efforts. Additionally, statistical analysis will identify relationships and spatial contamination patterns, providing a solid basis for targeted interventions and management strategies in Moatize. More broadly, this study enhances global understanding of contamination associated with coal mining, particularly in an African context, and supports international efforts toward sustainable development.

## Study area

**Fig. 1:**
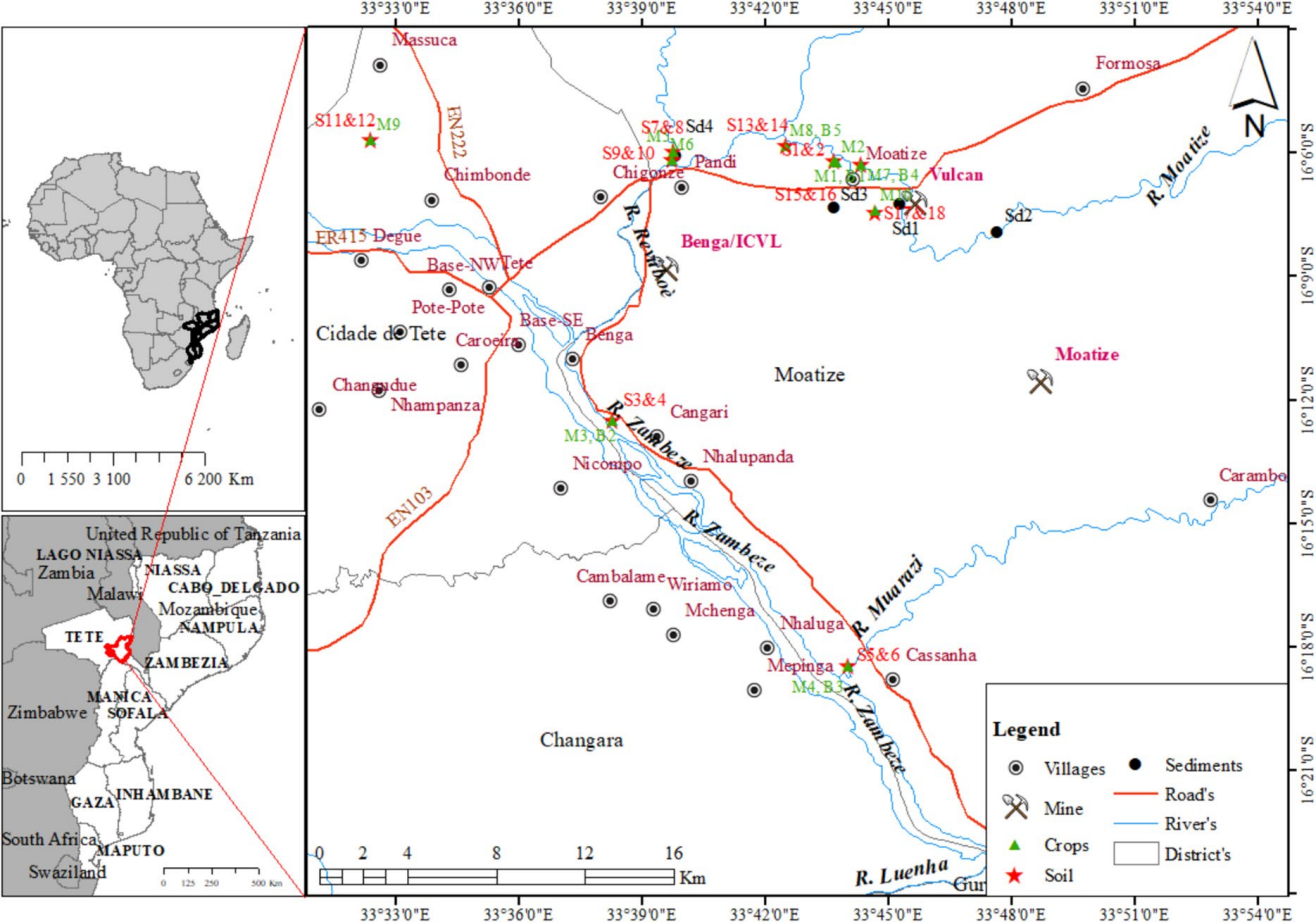
Location map of the Moatize district, showing sampling points (sediments, soils, and plants), infrastructure, and settlements (modified from Cossa et al., 2026)

The Moatize district, situated in central Mozambique’s Tete Province (15˚ 37’ – 16˚ 38’ S, 33˚ 22’ – 34˚ 28’ E), covers 8,462 square kilometers and had a population of 343,546 according to the 2017 census (INE, 2023; Fig. 1). Moatize experiences a local steppe climate with low annual rainfall. The average yearly temperature is 26.5 °C, but it can reach as high as 45 °C (Ferrier & Ruppel, 2025). Geologically, the district is partly within the extensive Gneissic Granitic Complex of the Mozambique Belt, which features prominent "Inselbergs" formed by post-Karroo intrusive rocks (MAE, 2014).

The Moatize Coal Basin is a graben, about 35 km long and 2 km wide. Part of the east–west-trending Zambezi Basin, it contains the coal-bearing Karoo Supergroup, which rests unconformably on Proterozoic basement rocks (Vasconcelos, 2000). The Supergroup is divided into four main stratigraphic units: the Vúzi, Moatize, Matinde, and Cádzi Formations (Vasconcelos, 2012). The bituminous coal in the Moatize Formation has low to medium volatile content (Fernandes et al., 2015). Within the Moatize coalfield, the economically productive series consists of six coal complexes arranged from bottom to top: Souza Pinto, Chipanga, Bananeiras, Intermédia, Grande Falésia, and André (Vasconcelos, 2012).

The main economic drivers of the district are agriculture, livestock, and mining. Agriculture is the leading sector, with nearly all households engaging in small-scale, manual, and rain-fed intercropping of local varieties (INE, 2023). However, production is often interrupted by the high risk of crop failure due to poor soil moisture retention during the growing season. To address these issues, some families use traditional soil fertilisation methods, such as fallowing or adding plant residues, manure, and ash (MAE, 2014).

Large-scale open-pit coal mining in Moatize is a major source of geochemical contamination in local soils and sediments (Marove et al., 2022). Through AMD and coal dust dispersal, mining activities release PTEs into the environment (Chandamela, 2021). These pollutants disrupt the local agricultural system, especially subsistence crops such as maize (*Zea mays*) and cowpea (*Vigna unguiculata*), where PTE accumulation reduces yields and threatens both food security and human health (Li et al., 2025).

Environmental degradation is worsened by unregulated local practices, especially the disposal of fly ash from coal-fired brick kilns. This waste introduces PTEs and alters soil physicochemical properties, ultimately reducing fertility (Saha et al., 2021). The combination of industrial mining and these uncontrolled community practices has led to ongoing, widespread pollution. Although limited research indicates that small amounts of coal ash might provide minor soil-conditioning benefits (Meshram et al., 2025), the unregulated disposal common in Moatize causes serious ecological damage (Yadav et al., 2021). Such degradation likely contributes to the increasing morbidity rates observed in the local population (Eggar et al., 2021).

## Materials and methods

### Sampling of sediments, soils, and food crops

This research utilised a rigorous stratified purposive sampling approach to study how PTEs transfer from contaminated soils to food crops, and the risks to the food chain. Eleven coal samples were collected, including three (about 5 kg each) from accessible outcropping layers used by local communities, and eight from run-of-mine (ROM) materials supplied by mining companies. To analyse contamination patterns related to agriculture, four top sediment samples (0–10 cm) were taken from floodplains where contaminants tend to settle. Additionally, 18 composite soil samples were gathered from nine farm locations at two depths (0–30 cm and 30–60 cm). This dual-depth method helps identify contamination sources: higher PTE levels in the topsoil than in the subsoil point to surface pollution from sources such as coal dust, contaminated irrigation water, and runoff, while subsoil concentrations reflect natural geogenic levels and long-term leaching. To directly measure PTEs uptake, 15 staple food crops were collected from the same farms: ten Zea mays and five cowpeas. The sampling plan aimed to capture a complete geochemical profile of the Moatize agricultural landscape. Sampling locations were selected based on proximity to active mining and importance to local food production. Because Moatize’s agriculture depends on seasonal rainfall and river water for irrigation, samples included floodplain sediments and farm soils to assess contaminant transfer through water and sediment. To compare different mining intensities, sites were divided into proximate (≤ 500 m) and distal (> 500 m) zones. Following standard procedures (de São, 2011), all samples were bagged and immediately sent to the Bureau Veritas laboratory for analysis.

### Laboratory analysis and quality control

To preserve sample integrity and prevent degradation, soil and food crop samples were first air-dried and then oven-dried at 40 °C at the Bureau Veritas laboratory. Each sample was individually ground into a fine powder and homogenised; all processing equipment was carefully cleaned between samples to avoid cross-contamination.

For chemical analysis, samples were transported to the Bureau Veritas laboratory in Pretoria. Cation concentrations were measured by ICP-MS, while anions were analysed by ion chromatography (IC). These methods were selected for their high sensitivity and accuracy, ensuring reliable geochemical data. Sample preparation protocols were tailored for each analysis type. For metal analysis, soil and plant samples were digested with a mixture of HNO₃, H₂SO₄, and HClO₄ (Standard Method; SM 3030 F). For anion analysis, an aqueous extraction method was used for soil and sediment samples. Additionally, soil pH and electrical conductivity (EC) were measured with a calibrated soil analyser in accordance with SM 4500 H + B and 2510 B, respectively.

To ensure data reliability, analytical methods were strictly controlled using Certified Reference Materials (CRMs), specifically SM 1640 for cations and SM 4500 for anions, to verify accuracy and precision. To minimise error and assess consistency, replicate analyses were performed on both samples and reference materials. Results indicated that percentage recoveries for all analytes, including Al, As, Ba, Cr, Cu, Fe, Mn, Mo, Ni, S, V, Zn, NO₃⁻, and F⁻, fell within the acceptable range of 80–120%. Furthermore, the calibration curves showed high linearity, with R^2^ values exceeding 0.99, providing high confidence in the measurements. The limit of detection (LOD) and limit of quantification (LOQ) for each substance are provided in Table 1.

**Table 1:** Quality assurance and quality control (QA/QC) results for PTEs

	%R	R^2	LOD (mg/kg)	LOQ (mg/kg)
Al	98.80	0.9999	1.70E-02	5.15E-02
As	101.39	0.9999	3.00E-03	9.09E-03
Ba	100.11	0.9998	1.00E-03	3.03E-03
Cr	94.35	0.9995	1.00E-02	3.03E-02
Cu	97.60	0.9999	2.00E-03	6.06E-03
Fe	99.14	0.9998	3.00E-03	9.09E-03
Mn	96.52	0.9999	1.00E-03	3.03E-03
Mo	92.16	0.9998	1.00E-03	3.03E-03
Ni	99.48	0.9999	1.00E-03	3.03E-03
S	107.27	0.9987	4.20E-02	1.27E-01
V	97.83	0.9998	1.00E-03	3.03E-03
Zn	85.35	0.9999	1.00E-03	3.03E-03
NO₃⁻	91.50	0.9991	1.00E-01	3.03E-01
F^−^	99.03	0.9999	1.00E-01	3.03E-01

## Data analysis

### Soil and sediment pollution indices

To evaluate the extent of environmental impact, soil and sediment properties were compared against established international standards (FAO/WHO, 2006; Canadian Council of Ministers of the Environment (CCME), 2007; NRCS, 2007; Chen et al., 2021). Furthermore, eight indices were calculated to assess the ecological risk and contamination levels of PTEs: the geo-accumulation index (Igeo), Enrichment Factor (EF), Contamination Factor (CF), pollution load index (PLI), contamination degree (CD), ecological risk index (Er), toxic unit (TU), and toxic risk index (TRI) (Table 2). These calculations used the geochemical background values set by Wedepohl (1995) for the Upper Continental Crust (UCC). The use of global UCC values is justified by the current lack of officially established local geochemical baselines for the Moatize area. By applying these widely accepted standards, the study ensures international comparability and offers a consistent, conservative baseline for assessing human-related enrichment in similar geological contexts.

**Table 2:** Classification of pollution indices from sediments and soils in Moatize

Index	Categories	Description	References
Igeo	Igeo ≤ 0	Uncontaminated	Ali et al. (2023); Zhang et al. (2021)
	0 < Igeo ≤ 1	Uncontaminated to moderately contaminated	
	1 < Igeo ≤ 2	Moderately contaminated	
	2 < Igeo ≤ 3	Moderately to heavily contaminated	
	3 < Igeo ≤ 4	Heavily contaminated	
	4 < Igeo ≤ 5	Heavily to extremely contaminated	
	5 < Igeo	Extremely contaminated	
	EF < 2	Low enrichment	Gopal et al. (2023); Shirani et al. (2020)
EF	2 ≤ EF < 5	Moderate enrichment	
	5 ≤ EF < 20	Significant enrichment	
	20 ≤ EF < 40	Very high enrichment	
	EF ≥ 40	Extremely high enrichment	
CF	CF < 1	low contamination	Hakanson (1980)
	1 ≤ CF < 3	Moderate contamination	
	3 ≤ CF < 6	Considerable contamination	
	CF > 6	Very high contamination	
PLI	PLI < 1	no pollution	Gopal et al. (2016)
	PLI > 1	pollution	
Cd	Cd < 8	Low degree of contamination	Sahoo et al. (2016)
	8 ≤ Cd < 16	Moderate degree of contamination	
	16 ≤ Cd < 32	Considerable degree of contamination	
	Cd > 32	Very high degree of contamination	
TRI	TRI ≤ 5	no toxic risk	Bembamba & Sako (2025)
	5 < TRI ≤ 10	low toxic risk	
	10 < TRI ≤ 15	moderate toxic risk	
	15 < TRI ≤ 20	considerable toxic risk	
	TRI > 20	very high toxic risks	
TU	TU < 4	low toxicity	Sabbagh et al. (2024); Pedersen et al. (1998)
	4 ≤ TU ≤ 6	moderate toxicity	
	TU > 6	heavy toxicity	

#### _Geoaccumulation index (Igeo)_

The geoaccumulation index is a geochemical parameter proposed to evaluate soil pollution levels (Muller, 1969). Igeo is calculated using Eq. (1).

1 $$\mathrm{Igeo}={\mathrm{log}}_{2}\left(\frac{Cn}{1.5 \times Bn}\right)$$

Here, Cn denotes the measured elemental concentration in the sediment, while Bn is the geochemical background value of the Earth’s crust. The constant 1.5 is used to account for potential variations in background values caused by natural lithogenic processes.

#### _Enrichment Factor (EF)_

The Enrichment Factor helps estimate the amount of metal in sediments and differentiate between natural and human-made sources. Because of their high abundance in the Earth’s crust, iron (Fe) and aluminium (Al) are common reference elements for normalisation (Gopal et al., 2023). In this study, EF was calculated using Eq. (2), with Fe chosen as the normalising element.

2 $$EF= \frac{\left(\frac{Metal}{Fe}\right)Sample}{\left(\frac{Metal}{Fe}\right)Background}$$

Here, $\left(\frac{Metal}{Fe}\right)Sample$ represents the ratio of the element concentration to the reference element concentration in the sample, while $\left(\frac{Metal}{Fe}\right)Background$ is the equivalent ratio in the geochemical background. The EF results are classified into five contamination categories, as detailed in Table 2.

#### _Contamination factor (CF)_

The Contamination Factor, as introduced by Hakanson (1980), was used to assess the level of contamination by individual elements in soils. The CF for each element was calculated using Eq. (3).

3 $$CF= \frac{{C}_{metal}}{{C}_{Background}}$$

Here, C_metal_ indicates the measured concentration of the metal in the sediment sample, whereas C_Background_ is the geochemical background level for that specific element.

#### _Pollution load index (PLI)_

The Pollution Load Index evaluates the overall metal contamination in sediment samples (Ali et al., 2023) and is calculated using Eq. (4).

4 $$\mathrm{PLI}= \sqrt[\mathrm{n}]{{\mathrm{CF}}_{1 }\times {\mathrm{CF}}_{2 }\times {\mathrm{CF}}_{3 }\times \dots \times {\mathrm{CF}}_{\mathrm{n }}}$$

Here, n is the total number of metals studied, and CF indicates the contamination factor for each element.

#### _Contamination degree (CD)_

The Contamination Degree measures the total pollution level at a particular sampling site (Hakanson, 1980) and is calculated using Eq. (5).

5 $$\mathrm{Cd}=\sum_{\mathrm{i}=1}^{\mathrm{n}}{\mathrm{CF}}_{\mathrm{i}}$$

Here, n represents the number of metals analysed, and CF indicates the contamination factor for each element.

#### _Ecological risk index (Er)_

The potential ecological risk of individual contaminants was assessed using the ecological risk index (Er), calculated according to Eq. (6) (Hakanson, 1980)

6 $$\mathrm{Er}=\mathrm{Tf}\times \mathrm{CF}$$

The toxic response factor (Tr) measures each element’s inherent toxicity. The specific values used in this assessment, sourced from Tisha et al. (2020) and Chandra et al. (2024), are 1 for Fe, Mn, and Zn; 2 for Cr; 5 for Co, Cu, Pb, and Ni; and 10 for As. The resulting Er values were categorised as: Er < 40 low pollution; 40 ≤ Er < 80 moderate pollution; 80 ≤ Er < 160 considerable pollution; 160 ≤ Er < 320 high pollution; and Er ≥ 320 very high pollution (Chandra et al., 2024).

#### _Toxic unit (TU) and toxic risk index (TRI)_

To evaluate the ecological consequences of soil contamination by PTEs, this study utilised the Toxic Unit (TU) and the Toxicity Risk Index (TRI). Calculations were based on the measured concentrations of five specific PTEs: Cu, Cr, Ni, Pb, and Zn.

The TU is the ratio of a pollutant’s concentration to its corresponding probable effect level (PEL) (Pedersen et al., 1998). To characterise the potential acute toxicity of the combined pollutants at a specific site, the sum of TUs is calculated using Eq. (7) (Pedersen et al., 1998; Sabbagh et al., 2024):

7 $$\mathrm{TU}={\sum }_{i=1}^{i=n}TUi={\sum }_{i=1}^{i=n}\frac{Ci}{PEL}$$

Here, Ci is the measured concentration of an individual metal, and PEL is the Probable Effect Level, defined as the chemical concentration threshold above which adverse biological effects are likely to occur (Pedersen et al., 1998). The PEL values used in this study were adopted from MacDonald et al. (2020).

While TUs provide a snapshot of acute risk, they may underestimate the broader ecological toxicity of heavy metals by failing to account for the Threshold Effect Level (TEL) (Bembamba & Sako, 2025). To provide a more holistic assessment, this study employed the TRI as proposed by Zhang et al. (2016). The TRI integrates both TEL and PEL benchmarks (MacDonald et al., 2020; Wu et al., 2022), with the specific index for each element (TRIi) determined through Eq. (8).

8 $$\mathrm{TRIi}=\sqrt{\frac{\left({\left(\frac{\mathrm{Ci}}{\mathrm{TEL}}\right)}^{2}+\left({\left(\frac{\mathrm{Ci}}{\mathrm{PEL}}\right)}^{2}\right)\right)}{2}}$$

The Integrated Toxicity Risk Index (TRI) for a single sample is then determined by summing the individual risk indices of all measured metals (Eq. 9):

9 $$\mathrm{TRI}={\sum }_{\mathrm{i}=1}^{\mathrm{n}}\mathrm{TRIi}$$

where Ci is the measured content of each heavy metal and n is the total number of metals analysed. The calculated values for both the sum TU and TRI are categorised according to the risk levels defined in Table 2.

### Bioaccumulation factor and human health risk assessment

#### Bioaccumulation factor (BAF)

The transfer of PTEs from soil to plants is a crucial food safety issue (Li et al., 2020). This study uses the bioaccumulation factor (BAF) to assess PTE uptake and movement in plants (Dahmani-Muller et al., 2000). Calculated based on Raskin et al. (1994), the BAF measures the bioavailability and buildup of PTEs from soil into plant tissues (Eq. 10), acting as an important indicator of contaminant behaviour.

10 $$\mathrm{BAF }= {\mathrm{C}}_{\mathrm{cp}} /{\mathrm{C}}_{\mathrm{soil}}$$

Here, Ccp indicates the concentration of PTEs in the crop, and Csoil represents the concentration of PTEs in the soil (both measured in mg/kg). A BAF value below 1 suggests limited mobility and sequestration of heavy metals in the soil. Conversely, a BAF greater than 1 indicates effective plant uptake and accumulation of PTEs relative to the surrounding soil (Sharma et al., 2018).

#### Non-carcinogenic risk

The potential health risks from consuming locally sampled food contaminated with PTEs were assessed using established quantitative indices. Non-carcinogenic risks were evaluated using the Estimated Daily Intake (EDI), Hazard Quotient (HQ), and Hazard Index (HI), while cancer risk was assessed using the Carcinogenic Risk (CR) index.

#### Estimated daily intake (EDI)

The Estimated Daily Intake (EDI) of PTEs was calculated using Eq. (11), following the methodology established by Atta et al. (2023).

11 $$\mathrm{EDI }=\text{ Cp x Cf x Dfi}/\mathrm{Bw}$$

Here, Cp indicates the concentration of PTEs in plants (mg/kg), Cf is the fresh-to-dry weight conversion factor (0.085; Atta et al., 2023), Dfi is the daily average food intake, and Bw is the average body weight. The parameters used in this study are shown in Table 3.

**Table 3:** Exposure parameters and input values used for the estimation of non-carcinogenic and carcinogenic health risks in adults and children within the Moatize district

Parameter	Symbol	Unit	Adult	Child	Source
Daily average food intake	Dfi	kg/person/day	0.6	0.0265	UNDP (2019)
Ingestion rate (soil)	IRsoil​	mg/day	100	200	US Epa (2011)
Ingestion rate (crop)	IRcrop​	g/day	300	150	FAO/WHO (2011)
Exposure frequency	EF	days/year	350	350	Study Area
Exposure duration	ED	years	30	6	US Epa (2011)
Body weight	BW	kg	70	15	US Epa (2011)
Averaging time (non-carc)	ATnc​	days	10,950	2,190	ED × 365
Averaging time (carc)	ATc​	days	25,550	25,550	70 years × 365

#### Hazard quotient (HQ)

To evaluate non-carcinogenic health risks, the Hazard Quotient (HQ) was calculated for adults and children at each site using Eq. (12).

12 $$\mathrm{HQ}=\mathrm{EDI}/\mathrm{RfD}$$

Here, RfD stands for the oral reference dose for each element. The values used are Cr (1.5), Cu (0.04), Fe (0.7), Ni (0.02), Mn (0.014), and Zn (0.3) mg/kg bw/day (US-EPA IRIS, 2006).

#### Hazard index (HI)

The Hazard Index (HI) was determined by summing the individual Hazard Quotients (HQ) for all examined PTEs using Eq. (13) (US Epa, 2002).

13 $$\mathrm{HRI}={\sum }_{n=1}^{\infty }\left(\mathrm{HQ}\right)$$

An HQ or HI value less than 1 indicates that the non-carcinogenic health risk remains within acceptable limits; on the other hand, values exceeding 1 suggest a potential for adverse health effects among consumers (US Epa, 2019).

#### Carcinogenic risk (CR)

The Carcinogenic Risk indicates the estimated likelihood of developing cancer over a lifetime due to ongoing exposure to specific carcinogens in food. This risk is assessed using ingestion cancer slope factors (CSF), which estimate the increased probability of developing cancer over a 70-year lifespan. According to Mulware (2013), the International Agency for Research on Cancer (IARC) classifies As, Cr, and Ni as human carcinogens. The corresponding ingestion slope factors mg/kg/day^−1^ used in this study are: As (1.5), Cr (0.5), Ni (0.00084). Calculations for CR were conducted separately for adults and children, as shown in Eq. (14). Notably, Cu was excluded from the carcinogenic assessment because it is classified by the US EPA as group D (not classifiable as to human carcinogenicity); however, its non-carcinogenic effects were fully evaluated through the HQ to ensure a complete risk profile. Calculations for CR were performed separately for adults and children to account for differences in body weight and ingestion rates.

14 $$\mathrm{CR}=\text{ EDI X ICSF}$$

In this assessment, CR stands for the carcinogenic risk, EDI indicates the estimated daily intake, and CSF refers to the ingestion cancer slope factor. A CR value between 10^–6^ and 10^–4^ indicates an acceptable range for predicted lifetime cancer risk. Therefore, risk factors below 10^–6^ are generally considered negligible, while those above 10^–4^ suggest a potential health concern (US Epa, 2019, 2020).

### Geospatial and statistical analysis

Descriptive statistics and spatial variation mapping (ArcGIS) were used to summarise the dataset and visualise PTE distribution. Due to the small sample size and non-normal distributions confirmed by Shapiro–Wilk testing (p < 0.05), nonparametric statistics were adopted, with central tendencies and variability reported as medians and ranges. Source identification was conducted using Pearson’s correlation, PCA/FA, and HCA in IBM SPSS (v. 20). While acknowledging the sample-to-variable ratio limitations, PCA/FA suitability was verified through KMO and Bartlett’s tests and utilized in a complementary, exploratory capacity. To ensure the stability of the identified geochemical signatures, results were cross-validated using HCA (Ward’s method and Squared Euclidean distance). This dual multivariate approach allowed for a robust distinction between anthropogenic and geogenic mechanisms within the specific geoenvironmental context of the Moatize region.

## Results

### Elemental composition of coal

Elemental concentrations in Moatize coal samples showed notable enrichment compared to the global hard coal benchmarks established by Ketris and Yudovich (2009). The average concentrations of Ba, Cr, Cu, Mn, Mo, V, and Zn all exceeded these global values (Table 4). Specifically, Ba, Cr, Mo, and V concentrations exceeded the benchmarks in all analysed samples. In contrast, higher concentrations of U, Cu, Zn, and Mn were observed in eight, five, five, and four individual samples, respectively. Conversely, the As concentration remained consistently below the established benchmark across all analysed samples (Table 4).

**Table 4:** Concentration of PTEs in coal from Moatize and comparison to Ketris and Yudovik (2009), in mg/kg

Element	C1	C2	C3	C4	C5	C6	C7	C8	C9	C10	C11	Mean	Ketris and Yudovik (2009)
Al	3834	3869	4684	4601	3975	2789	4033	3989	3974	4160	3636	3959	-
Fe	6233	6367	8656	8735	3351	2222	7725	5615	6870	4778	4647	5927	-
S	5408	5861	11,173	9237	6619	11,021	9268.9	6872	7912	7424	9619	8219	-
Si	31,271	31,092	15,127	30,664	34,881	18,725	24,576	26,928	27,896	30,603	24,796	26,960	-
As	4.0	4.0	2.2	6.1	1.4	1.0	8.36	4.16	2.39	3.29	1.59	3.5	9.0 ± 0.7
Ba	**233**	**230**	**254**	**420**	**234**	**151**	**198**	**234**	**246**	**338**	**252**	**253.7**	150 ± 10
Cr	**32.0**	**31.1**	**34.3**	**31.0**	**29.4**	**24.2**	**83.1**	**62.6**	**39.6**	**45.6**	**33.5**	**40.5**	17 ± 1
Cu	16.1	15.0	**20.2**	**21.3**	16.2	**18.2**	**34.0**	**23.8**	13.9	12.4	11.1	**18.2**	16 ± 1
Mn	57.3	58.0	**117.1**	**97.2**	42.0	13.4	45.1	70.7	**285.0**	**430.2**	25.6	**112.9**	71 ± 5
Mo	**3.0**	**3.2**	**3.3**	**3.3**	**3.1**	**3.1**	**3.6**	**3.3**	**2.6**	**3.2**	**2.7**	**3.1**	2.1 ± 0.1
Ni	16.1	17.2	15.0	17.3	11.1	12.1	14.5	12.8	10.3	14	10.3	13.5	17 ± 1
U	**2.1**	**2.3**	**2.3**	**2.6**	**2.0**	**2.4**	**3.1**	**3.0**	1.8	1.5	1.6	**2.2**	1.9 ± 0.1
V	**60.0**	**66.1**	**67.0**	**61.3**	**56.2**	**58.2**	**80.5**	**67.2**	**47.7**	**51.2**	**33.2**	**58.8**	28 ± 1
Zn	18.2	19.1	34.2	**44.0**	18.1	24.0	**29.6**	**29.9**	**49.2**	**49.5**	21.5	**30.6**	28 ± 2

C: sample identification

### Physicochemical properties of soil and sediments

Analysis of soil and sediment samples from Moatize revealed distinct physicochemical properties and levels of toxic elements that exceeded regulatory limits by a wide margin (Table 5). The elemental analysis revealed contrasting distribution patterns between the two matrices. In sediments, elements such as V (Median = 109.5 mg/kg) and pH (Median = 8.21) showed low spatial variability, indicating relatively homogeneous depositional environments. In contrast, significant heterogeneity was observed in the soil samples, particularly for Al and Fe. For instance, soil Al concentrations exhibited a highly skewed distribution (Median = 168,616.1 mg/kg), with a large standard deviation relative to the mean, highlighting the presence of localised extreme outliers. Similarly, NO₃⁻ in soil showed a wide range (2–710 mg/kg), with the median (179.8 mg/kg) providing a more robust measure of central tendency than the mean, which was heavily influenced by high-concentration samples.

**Table 5:** Statistical summary of PTEs in soils and sediments from Moatize. All concentrations are reported in mg/kg, and EC is in μS/m

Element	Sediments						Soil						Limit	Standard
	Min	Max	Mean	Median	SD	NSA	Min	Max	Mean	Median	SD	NSA		
Al	82,250	120,600	93,065	84,705	18,460.1	4	72,500	811,200	46,013	168,616.1	168,616.1	18	1.4	FAO/WHO (2006)
As	6.2	22.6	15.8	17.175	7.7	4	8.1	67.5	43.9	16.06975	16.1	18	0.2	
Ba	579.0	978	739.5	700.5	176.5	4	200	687.0	466.3	167.3584	167.4	18	100	
Co	39.2	69.5	52.9	51.435	12.9	4	19.2	46.3	31.9	40,700	7.6	18	10	
Cr	68.1	73.7	70.0	69.01	2.5	-	20.7	133	60.8	31.06	24.7	1	100	
Cu	33.4	81.2	57.1	56.98	22.8	4	11.2	46.3	28.7	58.585	9.9	10	10	
Fe	52,700	79,020	67,760	69,660	12,400.9	4	26,600	152,700	64,044	29.005	27,904.4	18	10	
Mn	793.0	1017	887	869	97.1	4	288	1221	762.7	62,200	231.6	18	0.2	
Pb	8.9	43.7	26.3	26.3	24.6	1	2.9	79	13.6	716	16.9	9	10	
Zn	83.9	108.1	90.5	85.01	11.7	4	17.9	87.3	49.9	95.44	18.1	6	50	
V	107.0	117.2	110.8	109.5	4.3	3	9.1	215	98.4	10.33	44.7	3	108	Chen et al (2021)
U	49,220	74,535	62,113	62,347.5	6371.7	4	bdl	bdl	bdl	-	-	-	13	CCME (2007)
F-	11	17	13.6	13.6	3.0	3	bdl	bdl	bdl	-	-	-	0.3	WHO (1984); Environmental health criteria
NO3-	2.5	10	4.8	3.35	3.5	0	2	710	75.3	179.801	179.8	2	50	FAO (2006)
EC	7.9	16.9	12.5	12.5	3.7	4	4.8	61.6	18.7	12.55	15.3	18	4.0	NRCS (2007)
pH	8	8.6	8.3	8.215	0.3	4	7.2	8.7	8.1	7.99	0.4	14	6.5–7.5	FAO (2006)

*NSA* number of samples above limit, *bdl* below detection limit (U < 0.4 mg/kg; F < 0.1 mg/kg); *SD* standard deviation

Sediment samples showed higher pH values (8.0–8.6) than soil samples, with all readings surpassing the FAO/WHO (2006) limit of 7.5. Overall, 14 soil samples exceeded this threshold, with pH levels ranging from 7.2 to 8.7; notably, the highest pH values were found in the deeper soil layers. EC in all sediment and soil samples surpassed the NRCS (2007) limit of 4 μS/m. Soil EC values ranged from 4.8 to 61.6 μS/m, while sediment EC ranged from 7.9 to 16.9 μS/m. Unlike the pH trend, surface soils showed higher EC levels than deeper soils (Table 5).

Anions: Fluoride (F⁻) concentrations in sediments ranged from 11 to 17 mg/kg, with three samples exceeding the WHO (1984) Environmental Health Criteria. In contrast, F⁻ remained below the detection limit (< 0.1 mg/kg) in all soil samples. Nitrate (NO₃⁻) levels in sediments were within the FAO (2006) safety standards. However, two soil samples exceeded the 50 mg/kg threshold, reaching concentrations of up to 710 mg/kg. Surface soils contained higher NO₃⁻ levels than deeper soil layers (Table 5), similar to the trends observed for EC.

Concentrations of Al, As, Ba, Co, Fe, and Mn in all sediment and soil samples exceeded the FAO/WHO permissible limits (Table 5). Soils generally showed higher concentrations of Al, As, Fe, and Mn than sediments. Specifically, Al concentrations in soils ranged from 72,500 to 811,200 mg/kg, compared to 82,250 to 120,600 mg/kg in sediments. Arsenic levels in soil ranged from 8.1 to 67.5 mg/kg, while sediment concentrations ranged from 6.2 to 22.6 mg/kg. Similarly, Fe concentrations in soil (26,600–152,700 mg/kg) spanned a wider, mostly higher range than those in sediment (52,700–79,020 mg/kg). Mn followed a similar pattern, with soil concentrations between 288 and 1,221 mg/kg and sediment levels between 793 and 1,017 mg/kg. Sediments also showed higher levels of Cu, Zn, and U, with all samples surpassing their respective safe limits. Cu levels ranged from 33.4 to 81.2 mg/kg in sediments, compared to 11.2 to 46.3 mg/kg in soils. Zn concentrations varied from 83.9 to 108 mg/kg in sediments versus 17.9 to 87.3 mg/kg in soils. U levels in sediments ranged from 49,220 to 74,535 mg/kg, but U remained undetectable in soil.

In contrast, soils showed the highest concentrations of Pb and V. Pb ranged from 2.9 to 79.0 mg/kg in soils and from 8.9 to 43.7 mg/kg in sediments, while V ranged from 9.0 to 215.0 mg/kg in soils compared with 107–117 mg/kg in sediments. Cr was also more concentrated in soils (20.7–133 mg/kg) than in sediments (33.4–81.2 mg/kg); notably, except for a single sample, all soil concentrations remained below the FAO/WHO permissible limit of 100 mg/kg. Regarding vertical distribution, Al, As, Ba, Cr, Mn, and V were generally more concentrated in deeper soil layers, whereas Co, Pb, and Zn showed higher levels in surface soils (Table 5).

### Spatial distribution maps of PTEs

**Fig. 2:**
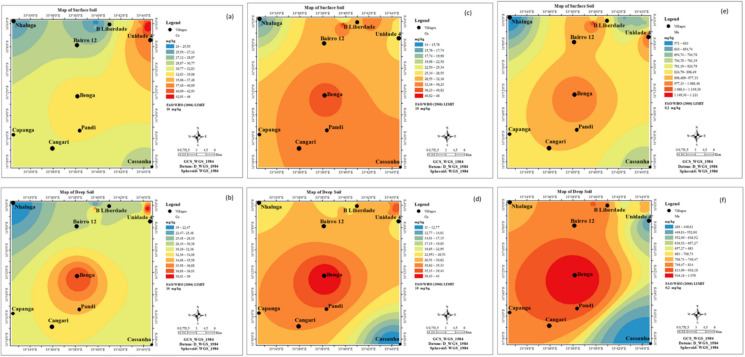
Spatial distribution maps of Co (**a**, **b**), Cu (**c**, **d**), and Mn (**e**, **f**) in both surface and deep soils from Moatize

**Fig. 3:**
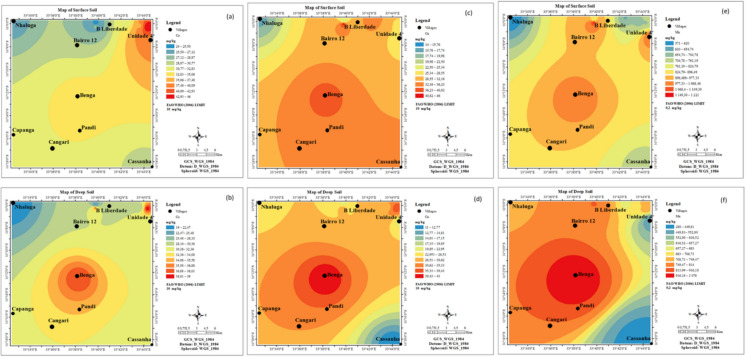
Spatial distribution maps of As (**a**, **b**), NO₃ (**c**, **d**), and EC (**e**, **f**) in surface and deep soil from Moatize

Spatial distribution maps (Figs. 2 and 3) illustrate the widespread elevation of PTE concentrations across both surface and deeper soil layers. Specifically, Co (Figs. 2a, b), Cu (Figs. 2c, d), and Mn (Figs. 2e, f) exhibited elevated concentrations in the northern and southern regions at both depths. Similarly, arsenic concentrations were highest in the southern region for both surface and deep soils (Figs. 3a, b). Most areas exhibited high NO₃⁻ concentrations across both soil layers (Figs. 3c, d). In contrast, soil EC displayed distinct spatial patterns: high levels were observed in the northwestern and southwestern parts of the deeper soils (Fig. 3e), whereas the highest surface soil EC was concentrated in the northern region (Fig. 3f).

### Pollution assessment and ecological implications for soils and sediments

**Fig. 4:**
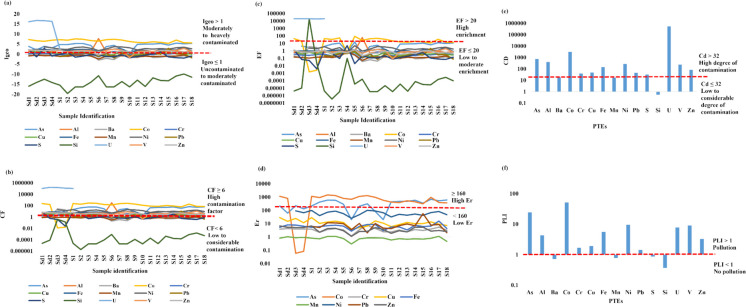
Pollution assessment indices for sediments and soils in Moatize. (**a**) Geoaccumulation index (Igeo); (**b**) Contamination factor (CF); (**c**) Enrichment factor (EF); (**d**) Ecological risk index (Er); (**e**) Contamination degree (CD); and (**f**) Pollution load index (PLI)

The pollution levels in Moatize sediments and soils showed significant contamination across a range of severities (Table S2; Fig. 4).

The Igeo evaluation (Table S1; Fig. 4a) revealed distinct contamination profiles for sediments and soils. Sediments were classified as uncontaminated by Mn (−0.73) and Ba (−0.47); moderately contaminated by Cr (0.41) and Pb (0.91); and moderately contaminated by Al (1.29), Cu (1.32), V (1.88), and Zn (1.91). Higher levels of pollution were observed for Fe (2.19), categorised as moderately to heavily contaminated, and for Ni (3.39) and As (3.23), which reached heavily contaminated status. Most significantly, sediments were extremely contaminated by Co (6.84) and U (16.47). Similarly, soils were uncontaminated by Mn and Ba (both −1.04) and moderately contaminated by Cr (0.11) and Cu (0.33). Soils reached moderate levels of contamination for Zn (1.08), V (1.54), and Al (1.65). Moderate to heavy contamination was noted for Fe (2.01), U (2.57), and Ni (2.86), while As (4.03) was classified as heavily contaminated. As with the sediments, soils were extremely contaminated with Co (6.53). This detailed breakdown demonstrates that while some elements remain within natural background ranges, others, specifically Arsenic, Cobalt, and Uranium, exhibit extreme levels of anthropogenic enrichment in both media.

Based on the EF analysis (Table S1; Fig. 4c), sediments showed minimal to low enrichment for As (−1.61), Al (0.55), Ba (1.69), Cr (0.30), Cu (0.61), Fe (1.0), Mn (0.13), Pb (0.07), V (0.82), and Zn (0.85). In contrast, Ni (2.69) exhibited moderate enrichment, while Co (15.65) showed significant enrichment. Most notably, U (19,936.9) displayed extremely high enrichment. In soils, low enrichment was observed for Ba (0.17), Cr (0.36), Cu (0.43), Fe (1.19), Mn (0.18), Pb (0.40), V (0.94), and Zn (0.68). Ni (2.29) showed moderate enrichment, while As (7.07) and Al (4.05) were significantly enriched. Co (29.54) indicated very high enrichment, and U (1,131.51) remained extremely highly enriched. The consistently elevated EF values for Uranium and Cobalt strongly suggest that their presence is driven by significant external anthropogenic sources rather than natural lithogenic background levels.

The CF values (Table S2; Fig. 4b) indicate varying levels of trace-element enrichment. In the sediments, results showed low Mn contamination (0.91), while Cr (2.00) and Pb (2.50) reached moderate levels. Significant contamination was noted for As (4.90), Al (3.72), Cu (4.00), V (5.54), and Zn (5.65). Additionally, Ba (12.05), Co (97.30), Ni (17.10), and U (13,800) displayed very high contamination levels. In contrast, soils exhibited low contamination from Mn (0.80) and moderate contamination from Cr (1.82) and Cu (2.60). Noteworthy contamination factors were observed for Ba (3.19), V (5.13), and Zn (3.94). Very high contamination levels in soils were recorded for As (30.88), Al (17.78), Co (9,143.00), Fe (6.57), Ni (12.84), and U (34,768.00). Overall, Cd showed extreme contamination in both sediments (6.75 × 10^6) and soils (5.54 × 10^5). The Pollution Load Index (PLI) further confirmed that both media are heavily polluted, with PLI values of 3.87 for sediments and 456.47 for soils.

The mean Ecological Risk (Er) index (Table S1; Fig. 4d) shows different levels of risk in sediments and soils. In sediments, results indicated a low ecological risk from Cr, Cu, Fe, Mn, Pb, and Zn. In contrast, Ni posed a moderate risk, while As showed a considerable risk. Notably, Co displayed a very high ecological risk in the sediment samples. In soils, Er values also indicated a low risk from Cr, Cu, Fe, Mn, Pb, and Zn, and a moderate risk from Ni. However, both As and Co exhibited very high ecological risk levels (Table S1), indicating more severe localised contamination in the soil profiles than in the sediments. Overall, the high CF, PLI, and Er indices show that contamination in the study area poses a serious ecological risk, with Cobalt and Uranium identified as the main pollutants of concern.

Sediment TUs ranged from 1.68 to 4.30, with most samples categorised as low toxicity. Conversely, soil samples exhibited a broader, more severe range (0.80–5.94), with 10 samples reaching moderate levels. Overall, soils demonstrated significantly higher toxic pressure than sediments across both indices. The TRI for both media followed the hierarchy: As > Cr > Ni > Cu > Pb > Zn. While sediment TRI peaked at "considerable" risk (16.11), soil TRI reached "very high" risk levels in 11 samples, highlighting a more critical ecological threat in the soil profile (Table 6).

**Table 6:** Toxic units (TU) and toxicity risk index (TRI) for sediments and soil samples in the Moatize region

Sample Id	TU	@	@	@	@	@	ƩTU	TRIi						TRI
@	@	@	@	@	@	@	@	As	Cr	Cu	Ni	Pb	Zn	@
Sd1	1.25	0.76	0.41	1.09	0.10	0.34	3.95	7.31	1.95	2.67	2.95	0.04	0.44	15.37
Sd2	0.36	0.82	0.22	0.00	0.01	0.27	1.68	0.62	2.29	0.74	0.00	0.00	0.28	3.93
Sd3	1.33	0.77	0.36	1.03	0.48	0.27	4.23	8.23	2.02	2.06	2.64	0.89	0.27	16.11
Sd4	0.77	0.76	0.17	0.74	0.01	0.27	2.71	2.74	2.00	0.45	1.35	0.00	0.27	6.81
S1	1.25	0.76	0.41	1.09	0.10	0.34	3.95	7.31	1.95	2.67	2.95	0.04	0.44	15.37
S2	2.46	0.43	0.15	0.44	0.08	0.12	3.68	28.09	0.64	0.35	0.49	0.02	0.05	29.65
S3	3.45	0.70	0.19	0.81	0.18	0.22	5.55	55.30	1.68	0.59	1.65	0.12	0.18	59.53
S4	3.43	0.82	0.22	0.97	0.17	0.23	5.84	54.66	2.28	0.76	2.37	0.12	0.19	60.38
S5	1.72	0.66	0.18	0.63	0.15	0.15	3.48	13.76	1.48	0.49	0.98	0.09	0.09	16.88
S6	0.12	0.23	0.06	0.28	0.05	0.06	0.80	0.06	0.18	0.05	0.20	0.01	0.01	0.52
S7	1.20	0.69	0.07	0.53	0.16	0.13	2.78	6.65	1.63	0.08	0.70	0.10	0.06	9.22
S8	1.84	1.48	0.10	1.11	0.03	0.13	4.69	15.67	7.45	0.14	3.10	0.00	0.07	26.43
S9	0.48	0.85	0.23	1.00	0.16	0.26	2.99	1.07	2.48	0.87	2.51	0.10	0.25	7.27
S10	0.12	0.48	0.16	0.74	0.07	0.19	1.75	0.06	0.78	0.39	1.37	0.02	0.13	2.76
S11	2.59	0.46	0.09	0.36	0.06	0.09	3.65	31.10	0.73	0.12	0.32	0.02	0.03	32.32
S12	2.75	0.64	0.12	0.56	0.11	0.15	4.34	35.20	1.42	0.24	0.79	0.05	0.09	37.78
S13	2.84	0.48	0.18	0.48	0.14	0.12	4.24	37.50	0.77	0.52	0.57	0.08	0.05	39.50
S14	3.42	0.70	0.14	0.58	0.11	0.28	5.24	54.53	1.67	0.32	0.85	0.05	0.29	57.71
S15	2.04	0.60	0.15	0.47	0.87	0.28	4.40	19.36	1.23	0.34	0.55	2.92	0.29	24.70
S16	3.97	0.76	0.20	0.75	0.12	0.14	5.94	73.37	1.99	0.62	1.42	0.05	0.07	77.53
S17	3.12	1.06	0.13	0.92	0.13	0.16	5.53	45.33	3.86	0.27	2.11	0.07	0.09	51.73
S18	3.55	0.62	0.11	0.53	0.03	0.13	4.97	58.52	1.33	0.18	0.69	0.00	0.06	60.79

*Sd* – sediments, *S* - soil

### Elemental composition of food crops

The comparison of co-located soils and crops revealed distinct patterns: cowpea (*Vigna unguiculata*) showed a higher bioaccumulation factor for Cr and Ni than *Zea mays*. Notably, 60% of all crop samples exceeded the FAO/WHO (2011) permissible limit of 0.1 mg/kg. The elemental composition of maize (*Zea mays*) and cowpea (*Vigna unguiculata*) revealed significant exceedances of the FAO/WHO (2011) guidelines. Generally, cowpea exhibited higher concentrations of PTEs than maize. As detailed in the average elemental concentration (Table 7), all samples of both crops exceeded FAO/WHO permissible limits for Al, Fe, and Mn. Furthermore, Cr (0.92–4.16 mg/kg) and Ni (0.47–1.18 mg/kg) surpassed their respective limits of 0.10 mg/kg in nine samples. In contrast, all Cu concentrations (0.88–2.07 mg/kg) remained well below the 5.00 mg/kg threshold. While Zn levels showed high variability, no specific FAO/WHO limits are established for this element. In contrast, all Cu concentrations (0.88–2.07 mg/kg) remained well below the 5.00 mg/kg threshold. The concentration of Cu in Cowpea showed high uniformity (Median = 1.23 mg/kg; Range: 1.14–1.34 mg/kg). This tight clustering suggests that Cu uptake in Cowpea is strictly regulated or that bioavailability was consistent across sampling sites, with all values remaining well below the FAO/WHO limit of 5 mg/kg. In contrast, Si levels in *Zea mays* demonstrated significant variability and a highly skewed, non-normal distribution (Median = 67.25 mg/kg; Range: 24.40–402.00 mg/kg). This wide range and the substantial gap between the median and the maximum value confirm that the distribution is heavily influenced by high-level outliers, likely resulting from localised differences in soil composition or variations in plant maturity at the time of sampling. Finally, although Zn levels showed high variability, no specific FAO/WHO limits have been established for this element.

**Table 7:** Statistical elemental concentration (mg/kg dry weight) of PTEs in crops from Moatize

	Zea Mays						Cowpea						FAO/WHO (2011)
	Min	Max	Mean	Median	SD	NSA	Min	Max	Mean	Median	SD	NSA	
Al	320,700	1,080,000	688,830	767,450	241,444	10	704,400	1,780,000	1,164,080	1,250,000	455,067	5	0.0003
Cr	0.15	1.34	0.54	0.15	0.52	10	0.917	4.16	2.14	1.53	1.28	5	0.1
Cu	0.10	2.07	1.18	1.11	0.72	0	1.14	1.34	1.23	1.22	0.08	0	5
Fe	218,900	2,140,000	1,063,880	1,022,600	606,207	10	1,550,000	3,340,000	2,456,000	2,270,000	847,838.4	5	0.11
Mn	6.89	20.71	13.699	13.28	4.16	10	16.32	29.88	25.61	28.81	5.64	5	0.2
Mo	0.05	0.338	0.1278	0.05	0.11	-	0.05	0.661	0.17	0.05	0.27	-	nd
Ni	0.15	0.708	0.3185	0.15	0.22	10	0.515	1.18	0.72	0.66	0.27	5	0.1
S	292	554	424.7	429.50	71.99	-	438.00	730.00	552.60	550.00	114.18	-	nd
V	0.10	0.42	0.29	0.32	0.12	-	0.526	0.749	0.66	0.66	0.09	-	nd
Zn	3.06	14.30	7.93	6.69	4.00	8	3.69	14.62	7.59	6.64	4.18	4	5
Si	24.40	402.00	102.51	67.25	109.41	-	62.60	138.00	94.52	95.20	30.96	-	nd

*nd*: no data; *SD*: standard deviation

### Source identification of PTEs in soil and crops

Following Cohen’s (2013) classification guidelines, the correlation matrix in Table 8 illustrates the relationships among the various elements.

**Table 8:** Pearson correlation analysis of elemental distribution in soil and between soil and associated crops from Moatize

	Al		As	Ba	Co	Cr	Cu	Ni	Pb	V	Zn	NO₃⁻	EC
Al	1												
As	−0.35		1										
Ba	0.16		−0.33	1									
Co	−0.09		−0.14	0.39	1								
Cr	−0.42		0.08	−0.05	0.01	1							
Cu	−0.30		−0.20	0.54**	0.44	0.16	1						
Fe	−0.33		0.26	−0.10	0.15	0.68^**^	0.10						
Mn	−0.51		0.01	0.15	0.32	0.57^**^	0.44						
Ni	−0.29		−0.03	0.25	−0.15	0.65^**^	0.49	1					
Pb	−0.12		0.04	0.19	−0.20	0.00	0.20	0.11	1				
V	−0.28		0.24	−0.11	0.08	0.84^*^	0.10	0.45	0.04	1			
Zn	−0.38		−0.30	0.54**	0.55^**^	0.30	0.77^**^	0.38	0.37	0.20	1		
NO₃⁻	−0.07		0.09	−0.33	−0.16	−0.18	0.05	−0.10	−0.01	−0.12	−0.16	1	
EC	−0.12		0.06	−0.22	−0.46	−0.18	−0.21	−0.20	−0.06	−0.15	−0.27	0.57^**^	1
pH	0.11		−0.23	−0.04	0.14	0.20	0.05	0.07	−0.35	0.39	0.12	−0.13	−0.11
	Al		Cr	Cu	Fe	Mn	Ni	V					
Al	1												
Cr	−0.41		1										
Cu	−0.44		0.09	1									
Fe	−0.33		0.70^**^	0.18	1								
Mn	−0.51		0.57^**^	0.69^**^	0.68^**^	1							
Ni	−0.38		0.83^*^	0.49	0.62^**^	0.83^*^	1						
V	−0.27		0.84^*^	0.05	0.90^*^	**0.53**	0.62^**^	1					
Zn	−0.44		0.27	0.65^**^	0.29	0.67**	0.50	0.17					

* Correlation is significant at *p* < 0.01

** Correlation is significant at *p* < 0.05

The correlation analysis of soil elements revealed strong positive associations (r ≥ 0.50) between several pairs, including Cu–Ba, Zn–Ba, Zn–Co, Fe–Cr, Mn–Cr, Ni–Cr, V–Cr, Zn–Cu, Mn–Fe, V–Fe, Ni–Mn, V–Mn, Zn–Mn, and EC–NO₃⁻. Notably, V–Co (r = 0.84) and V–Fe (r = 0.89) exhibited robust positive associations. Moderate positive correlations (r = 0.30–0.49) were identified for Co–Ba, Mn–Co, Cu–Co, Zn–Cr, Mn–Cu, Ni–Cu, Ni–Fe, Si–Fe, V–Ni, Zn–Ni, and Zn–Pb. In contrast, weak associations (r = 0.10–0.29) characterized the relationships between Ba–Al, pH–Al, Fe–As, V–As, Pb–Ba, Ni–Ba, Mn–Ba, Fe–Co, pH–Co, Cu–Cr, Pb–Cu, V–Cu, Zn–Fe, Pb–Ni, pH–S, Zn–V, and pH–V.

Regarding the relationship between soil and crops, strong positive correlations (r ≥ 0.50) were found for Fe–Cr, Mn–Cr, Ni–Cr, V–Cr, Mn–Cu, Zn–Cu, Mn–Fe, Ni–Fe, V–Fe, Ni–Mn, V–Mn, Zn–Mn, V–Ni, and Zn–Ni. Among these, Ni–Cr, V–Cr, and V–Fe exhibited particularly high coefficients, ranging from r = 0.83 to r = 0.90. Moderate positive correlations (r = 0.30–0.49) were observed for Ni–Cu and V–Si, while weak associations (r = 0.10–0.29) were noted between Zn–Cr, Fe–Cu, Zn–Fe, and Zn–V (Table 8).

The PCA accounted for approximately 80.4% of the cumulative variance for soil data and 88.2% for crop data (Table 9). The analysis focused on the first four principal components (PC1-PC4), which together accounted for most of the variance in both datasets. For the soil matrix, the four components explained 37.7%, 22.9%, 11.3%, and 8.5% of the variance, respectively, while in the crop matrix, they accounted for 46.0%, 22.5%, 10.9%, and 8.7%. Regarding the specific component loadings, PC1 in the soil was characterised by strong positive loadings for Ni, Cr, Mn, Cu, V, Fe, Co, S, EC, and As, whereas in crops, PC1 showed strong positive loadings for Mn, Ni, Cr, Fe, V, and Zn, but a strong negative loading for Al. For PC2, the soil exhibited strong positive loadings for S, Cu, and Zn, while correlating negatively with Si, Fe, and V; conversely, in crops, PC2 was positively loaded by Si, V, Fe, and Cr, and negatively by Cu, Zn, and S. PC3 in the soil was positively associated with Al, As, Co, and Pb, but negatively with EC, pH, and NO_3_^−^, while in crops, PC3 had a strong positive correlation with S and strong negative correlations with Al, Zn, Cu, and Ni. Finally, PC4 in the soil matrix was characterised by a prominent positive loading for Pb and a secondary positive loading for Zn, contrasted by negative loadings for Al and Co. In the crop matrix, PC4 showed positive correlations with Si and Cu and a strong negative correlation with Cr.

**Table 9:** Principal component analysis (PCA) of soil and crop elemental composition in the Moatize region

Variable	Soil				Crops			
	CP1	CP2	CP3	CP4	CP1	CP2	CP3	CP4
Al	−0.008	0.111	0.385	−0.436	−0.272	0.306	−0.353	0.192
As	−0.196	−0.311	0.336	−0.056	0.383	0.207	0.148	−0.42
Ba	0.239	0.253	0.156	0.123	-	-	-	-
Co	0.251	0.033	0.298	−0.372	-	-	-	-
Cr	0.349	−0.202	−0.043	0.11	-	-	-	-
Cu	0.274	0.321	−0.037	−0.143	0.254	−0.407	−0.236	0.441
Fe	0.243	−0.364	0.04	0.072	0.38	0.273	0.207	0.22
Mn	0.295	−0.192	−0.248	0.042	0.423	−0.136	−0.164	0.13
Ni	0.378	0.05	−0.105	−0.064	0.419	0.021	−0.194	−0.182
S	0.214	0.336	0.05	−0.128	−0.038	−0.314	0.726	0.359
Si	0.141	−0.409	0.159	0.001	0.024	0.515	−0.108	0.595
Pb	0.033	0.16	0.273	0.627	-	-	-	-
V	0.275	−0.351	−0.002	0.112	0.361	0.351	0.26	0.026
Zn	0.275	0.279	−0.067	0.298	0.29	−0.342	−0.289	0.099
NO3	−0.159	0.005	−0.341	−0.113	-	-	-	-
EC	−0.245	0.026	−0.407	0.052	-	-	-	-
pH	0.231	−0.056	−0.4	−0.29	-	-	-	-
Eigenvalues	6.409	3.887	1.919	1.452	4.605	2.252	1.089	0.875
% of Variance	37.7%	22.9%	11.3%	8.5%	46.0%	22.5	10.9%	8.7%
Cumulative %	37.7%	60.6%	71.9%	80.4%	46.0%	68.6%	79.5%	88.2%

**Fig. 5:**
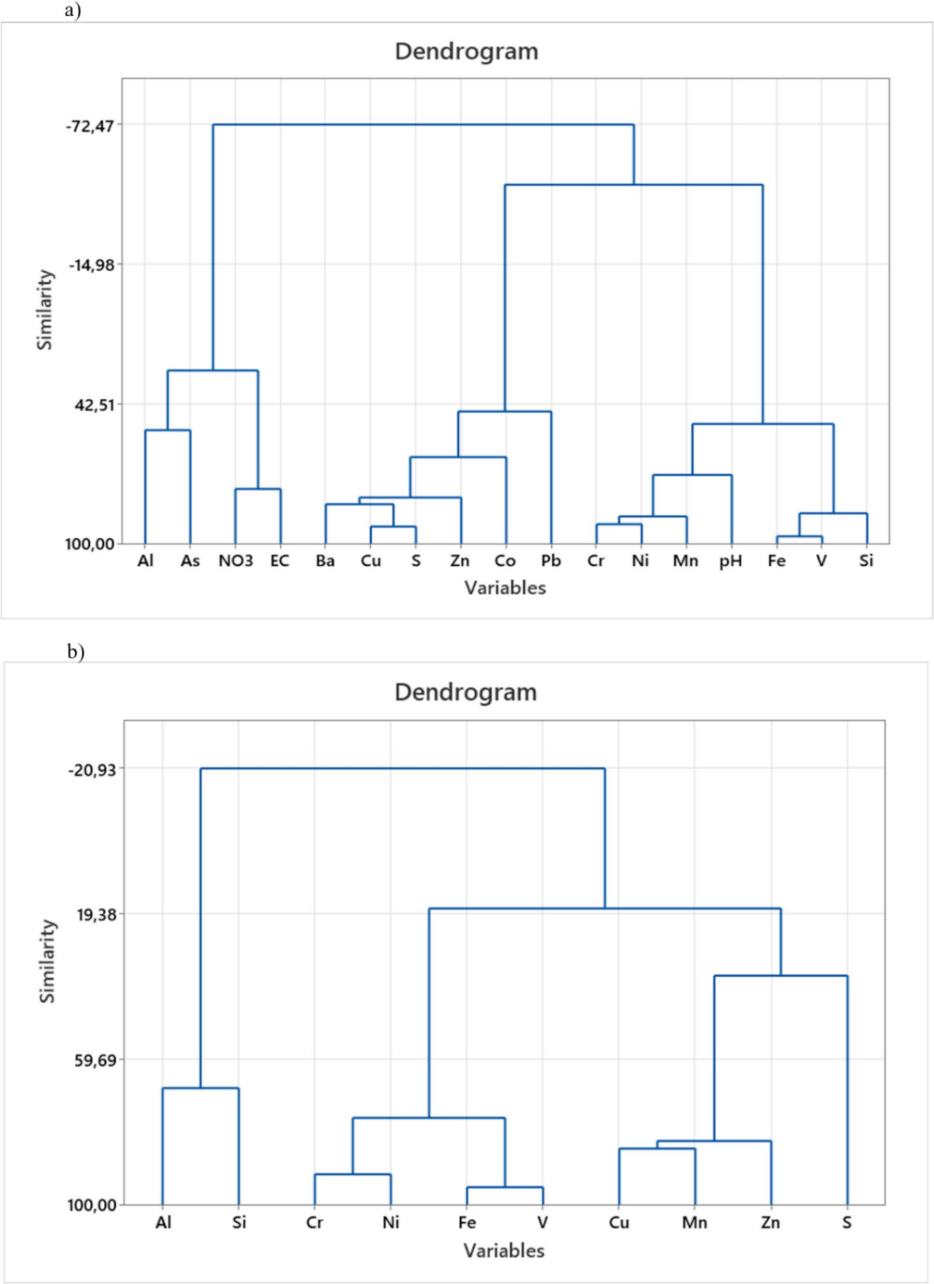
Hierarchical Cluster Analysis (HCA) dendrograms illustrating the elemental associations in (**a**) soil and (**b**) crops within the Moatize region

HCA using Ward’s linkage method categorized the elements based on similarities in their distribution patterns, resulting in four distinct clusters for soil and three for crops (Fig. 5). In the soil (Fig. 5a), Cluster 1 included Cu, S, Zn, Ba, Co, and Pb, while Cluster 2 consisted of Fe, V, Si, Cr, Ni, Mn, and pH. Cluster 3 grouped NO_3_^−^ and EC, and Cluster 4 represented a unique grouping of Al and As. In contrast, the crop data (Fig. 5b) yielded three clusters: Cluster 1 comprised S, Zn, Cu, and Mn; Cluster 2 comprised Fe, V, Ni, and Cr; and Cluster 3 comprised Al and Si.

### Bioaccumulation factors (BAF) of PTEs from soil to staple crops

The mean BAF values for the analysed food crops followed the descending order: Si (58.30) > Fe (24.71) > Al (8.61) > Mo (3.28) > S (2.14) > Zn (0.17) > Cu (0.05) > Ni (0.03) > Cr (0.03) > Mn (0.02) > V (0.01). Overall, cowpea exhibited higher BAFs than maize (*Zea mays*). Notably, concentrations of Cr, Mo, and Ni remained below the detection limit (BDL) in most maize samples (Table 10).

**Table 10:** Bioaccumulation factors (BAF) for common food crops harvested in Moatize

Crop number	Al	Cr	Cu	Fe	Mn	Mo	Ni	S	V	Zn	Si
Zm1	5.85	bdl	0.03	20.98	0.02	3.38	bdl	1.43	0.004	0.18	6.48
Zm2	3.72	bdl	bdl	9.31	0.01	bdl	bdl	1.18	bdl	0.14	5.37
Zm3	6.78	bdl	0.03	5.16	0.02	bdl	bdl	4.43	0.006	0.17	574.3
Zm4	6.40	bdl	bdl	3.40	0.02	bdl	bdl	1.28	0.002	0.06	7.18
Zm5	11.05	bdl	0.06	13.27	0.02	bdl	bdl	2.95	0.003	0.35	42.29
Zm6	10.98	bdl	bdl	19.62	0.01	bdl	bdl	1.02	0.003	0.04	2.92
Zm7	8.46	0.017	0.07	20.44	0.00	bdl	0.026	0.61	0.003	0.22	0.96
Zm8	9.77	0.027	0.05	64.46	0.02	2.25	0.033	0.89	0.005	0.26	0.64
Zm9	3.49	0.032	0.12	21.02	0.02	2.01	0.055	2.69	0.003	0.20	0.95
Zm10	4.22	0.010	0.07	5.86	0.01	2.14	0.014	4.44	bdl	0.14	0.12
C1	5.01	0.093	0.04	28.08	0.04	bdl	0.033	1.65	0.009	0.10	5.47
C2	9.90	0.041	0.03	32.99	0.02	6.61	0.040	3.50	0.007	0.08	197.1
C3	15.08	0.026	0.03	51.94	0.00	bdl	0.030	1.65	0.005	0.16	28.0
C4	20.51	0.024	0.02	45.82	0.04	bdl	0.031	0.97	0.007	0.24	1.73
C5	7.90	0.022	0.07	28.34	0.05	bdl	0.040	3.39	0.010	0.23	0.89
Mean BCF	8.61	0.032	0.05	24.71	0.02	3.28	0.034	2.14	0.005	0.17	58.29

*Zm*- Zea mays; *C*- Cowpea; *bdl*- below detection limit

### Assessment of daily metal intake (DMI) and human health risk

**Fig. 6:**
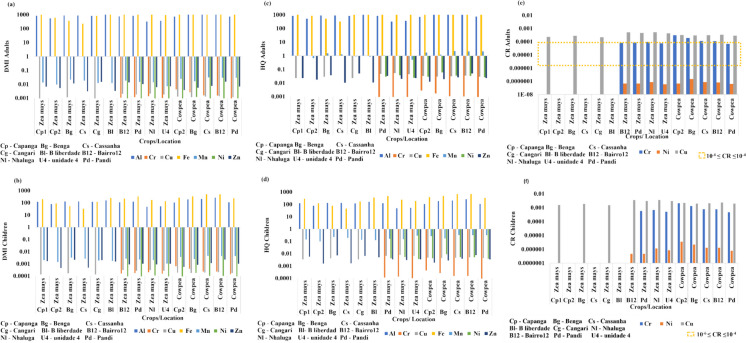
Human health risks from metal intake linked to Moatize crops. (**a**) and (**b**) Daily Metal Intake (DMI) for adults and children, respectively; (**c**) and (**d**) Hazard Quotient (HQ) for adults and children, respectively; and (**e**) and (**f**) Cancer Risk (CR) for adults and children, respectively. Sampling locations are abbreviated as follows: Cp – Capanga, Bg – Benga, Cs – Cassanha, Bl – B. Liberdade, Cg – Cangari, Nl – Nhaluga, B12 – Bairro 12, Pd – Pandi, and U4 – Unidade 4

The DMI for adults and children is summarised in Table S3 and Fig. 6 (a, b). For adults, DMI values ranged from 0.0005 to 1,558.50 mg/kg/day. The highest intake of potentially toxic elements (PTEs) followed this descending order: Fe (1,558.50) > Al (883.20) > S (0.48) > Si (0.10) > Mn (0.018) > Zn (0.008) > Ni (0.003) > Cr (0.0017) > Cu (0.0014) > V (0.0005). PTE intake was notably higher from cowpea consumption than from maize (Table S3; Fig. 6a-b). For children, DMI values ranged from 4.92E-05 to 229.4 mg/kg/day. Their intake followed a similar descending order: Fe (229.40) > Al (130.00) > S (0.07) > Si (0.014) > Mn (0.0026) > Cr (0.0025) > Zn (0.0011) > Cu (0.0002) > Ni (0.0001) > V (0.00007) > Mo (0.00005). Consistent with the adult data, children’s DMI reflected higher PTE levels in cowpea (Table S3; Fig. 6b).

The HQ for adults ranged from 0.0012 to 883.20 (Table S4, Fig. 6c), surpassing the safe threshold of 1.0 for Al and Fe across all crop samples. For Mn, the threshold was exceeded in *Zea mays* samples from Benga and Cassanha, as well as in all cowpea samples (Table S4, Fig. 6c). The THQ values followed the descending order: Fe (6,487.50) > Al (883.20) > Mn (1.28) > Cu (0.035) > Ni (0.033) > Zn (0.027). For children, HQ values ranged from 0.0002 to 327.80, with Al, Fe, and Mn frequently exceeding the safe limit of 1.0 (Table S4; Fig. 6d). The decreasing order of risk for children was Fe (327.80) > Al (130.00) > Mn (0.20) > Cu (0.0051) > Ni (0.0049) > Zn (0.0039).

The CR for the analysed elements followed a descending hierarchy, with Cr (9.03 × 10^–4^) exhibiting the highest risk, followed by Ni 1.34 × 10^–6^). These results indicate that the Cr risk exceeded the strict acceptable limit of 1.0 × 10^−4^ in several samples. For children, the mean CR was highest for Cr (9.03 × 10^−4^), followed by Ni (8.23 × 10^−8^). In all *Zea mays* and cowpea samples, the CR for Cr exceeded the acceptable limit for children. Furthermore, two *Zea mays* samples from Benga and Cangari exhibited a Cr-related risk that surpassed the standard acceptable range of 1.0 × 10^−6^ to 1.0 × 10^−4^ (Table S5; Fig. 6e, f).

**Fig. 7:**
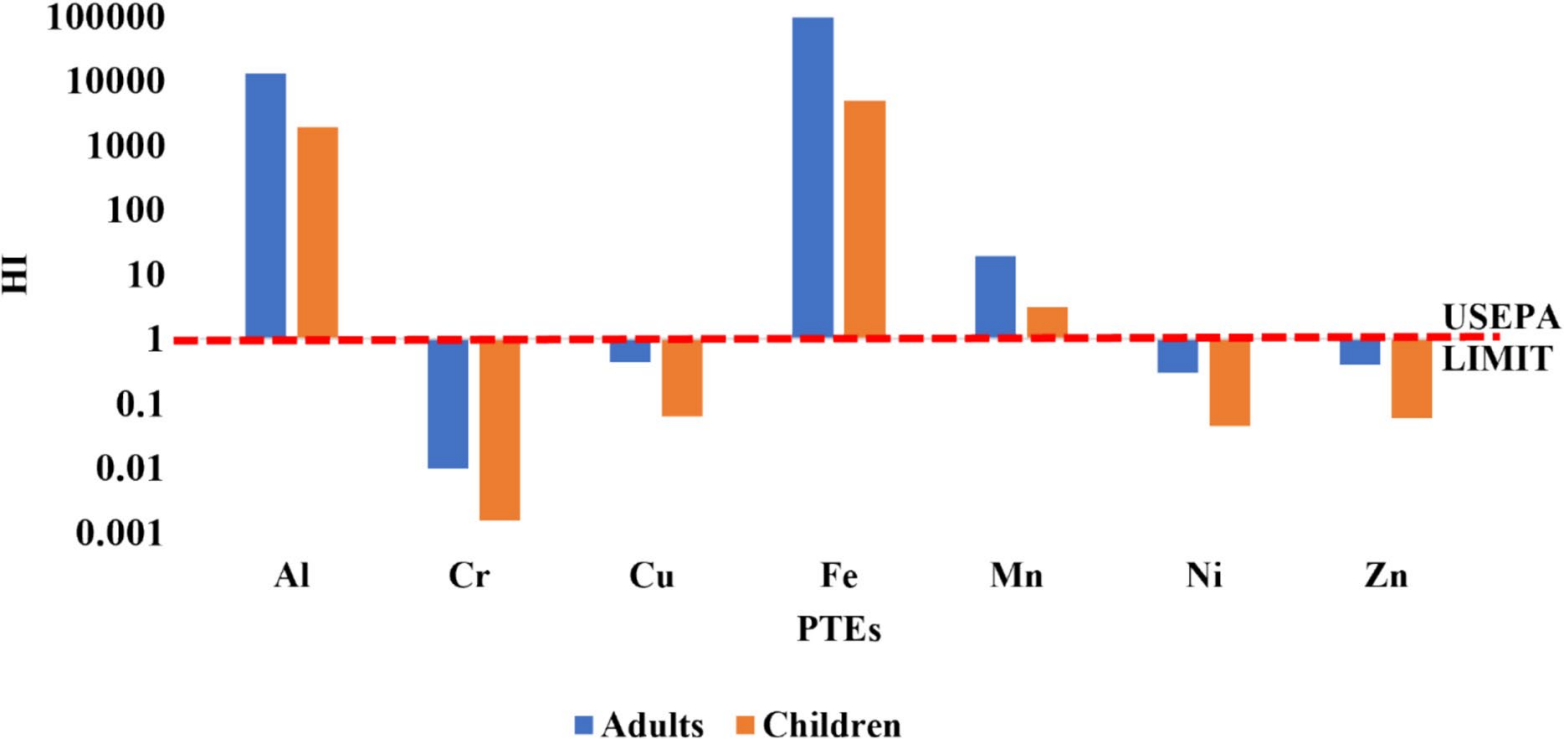
Hazard Index (HI) characterising the cumulative non-carcinogenic health risk from potentially PTEs for adults and children in the Moatize region

The HI, an overall non-carcinogenic risk indicator, for adults ranged from 0.010 to 97,313, with Cr, Cu, Ni, and Zn consistently below the safe threshold of 1. Similarly, children’s HI varied from 0.02 to 4916.6, also showing values less than 1 for Cr, Cu, Ni, and Zn (Fig. 7).

## Discussion

The Moatize study area presents critical environmental concerns due to naturally enriched local coal deposits. As documented by Habib and Khan (2021), these elevated trace elements pose significant risks throughout the coal life cycle, from extraction and processing to combustion, during which they are released as atmospheric pollutants. Consequently, the unique geochemical signature of this coal has resulted in severe and pervasive contamination, as evidenced by the degraded quality of the surrounding soil and sediment.

The environmental assessment confirmed that this contamination is widespread, as the physicochemical properties of the sediment and soil, including pH, EC, various anions, and PTEs, frequently exceed regulatory limits. This degradation of soil properties aligns with established findings in mining literature (Akbar et al., 2024; Kravchenko et al., 2025; Pandey et al., 2022). Specifically, the high pH observed in deeper soil profiles may reduce the solubility of certain metals while simultaneously increasing the mobility of oxyanions (Kicińska et al., 2022). Furthermore, high EC levels, indicative of dissolved salts originating from mining activities, impair plant growth (Rai et al., 2021). Similarly, elevated fluoride concentrations in the sediment pose significant risks to both aquatic and human health through bioaccumulation pathways (Kabir et al., 2020).

The mobility of PTEs in Moatize soils and sediments is influenced by the availability of elements and physicochemical properties. Elevated EC reflects high concentrations of dissolved ions, which enhance metal solubility through ion exchange (Binnemans & Jones, 2023). The presence of coal dust and ash reduces the soil’s buffering capacity (Liu et al., 2025). Oxidation of sulfide minerals, such as pyrite within coal, creates localised AMD conditions and lowers soil pH. This acidic environment transforms PTEs like Al, Fe, and Mn from stable phases into potentially bioavailable aqueous forms (Zhang et al., 2023). Such geochemical shifts account for high metal uptake even at distant sites, with fine coal dust acting as a persistent source of acidity that enhances metal mobility (Nieder & Benbi, 2024). Elevated PTE concentrations in soils and sediments across the region highlight a significant anthropogenic impact, posing environmental and health risks (Afahnwie et al., 2025d; Tiabou et al., 2025c). For example, a 2024 study by Rouhani et al. on European coal mining regions identified Zn, Pb, Mn, and Cr as major soil contaminants. These elements are released into the environment throughout the coal mining life cycle, from transportation and waste disposal to wastewater treatment. According to Jiang et al. (2020), these high concentrations threaten soil health and facilitate the entry of toxic elements into the food chain. This is consistent with other research on mining-related contamination. For instance, Wang et al. (2022) identified coal gangue and fly ash as the primary sources of leached PTEs in Northern Shaanxi’s coal mining areas, while Siddiqui et al. (2020) linked high PTE concentrations directly to India’s coal industry. Broadly, Rouhani et al. (2023b) also associate coal extraction and processing with increased environmental PTE burdens.

Spatial distribution maps illustrate widespread, elevated PTE concentrations across both surface and deeper soil horizons. These findings align with those of Masood et al. (2024), who reported highly concentrated PTE distributions near coal mining sites. Furthermore, Deng et al. (2022) indicated that while contamination is pervasive, coal mining operations typically exert a more severe environmental impact on surface layers.

Strong positive correlations (r ≥ 0.50) among PTEs in the soil indicate a common source, whether lithogenic, industrial, or mining-related, while weaker correlations suggest multiple origins (Li et al., 2020; Ahogle et al., 2023; Kachoueiyan et al., 2024b). A strong soil-to-crop correlation for Ni and Cr is a significant finding, as it signals substantial transfer into the food chain and poses a risk to consumer health (Chen et al., 2024).

PCA identifies a geogenic source through the co-loading of Fe, V, Cr, Ni, and Mn in both soil and crops. This grouping, combined with high Si levels in crops, reflects silicate mineral weathering (Zhou et al., 2025). This is consistent with studies identifying Cr, Ni, Cu, Mn, and V as geogenic constituents in soils (Ahogle et al., 2023; Li et al., 2024). Furthermore, the analysis indicates that crops naturally exclude Al at the root barrier (Sade et al., 2016), as evidenced by its negative correlation with other elements. Conversely, Pb is identified as the primary marker of anthropogenic input, typically linked to coal handling or atmospheric deposition (Chen et al., 2023; Jean-Lavenir et al., 2023; Simou et al., 2024). The grouping of Cu, S, and Zn suggests inputs from mining effluent or agricultural activities; specifically, the oxidation of pyritic coal generates acid mine drainage (AMD), which co-releases Cu and Zn (Zhang et al., 2021). These anthropogenic findings align with research identifying Pb, Zn, and Cu as key indicators of pollution (Chowdhury & Rahman, 2024; Yu et al., 2024; Yiika et al., 2023).

The resulting soil acidity from AMD is the dominant factor controlling element mobility. The inverse relationship between toxic elements (Al, As, Co, Pb) and pH/EC confirms that acidic conditions increase elemental solubility and subsequent plant uptake (Mensah & Amoakwah, 2024). Hierarchical Cluster Analysis reinforces this distinction by clearly separating pollution-related elements (Cu, S, Zn, Ba, Co, Pb) from geogenic elements. However, it also highlights that As mobility is uniquely complex and highly sensitive to redox conditions (Cheraghi et al., 2025).

Crop concentration patterns are shaped more by biological selectivity than by the simple abundance of elements in soil. The pronounced accumulation of Al and Fe in both plant species is attributed to the region’s geological enrichment in these metals and to the plants’ limited ability to exclude them at such high concentrations. In contrast, the marked enrichment of Cr and Ni in cowpea indicates a facilitated uptake mechanism. As a legume, cowpea releases organic acids to mobilise phosphorus (Chen et al., 2023); these acids also chelate Cr and Ni, inadvertently enhancing their uptake through the symplastic pathway (Garg et al., 2025). Meanwhile, Zea may’s fibrous root system acts as a physical barrier, sequestering elements like Al within the root cortex and restricting their transfer to the grain (Kaur et al., 2025). Contrasting statistical distributions further evidence this selective uptake. Cowpea exhibited high uniformity in Cu levels (Median = 1.23 mg/kg), with tight clustering suggesting regulated uptake or consistent bioavailability. In contrast, *Zea mays* showed a highly skewed Si distribution (Max = 402.00 mg/kg), highlighting the impact of localised outliers. These disparities mirror the soil matrix’s heterogeneity, where the median proved a more robust measure of central tendency than the mean for skewed parameters like Al and NO3^−^. Ultimately, cowpea’s stabilised accumulation suggests a more efficient, regulated process compared to the outlier-driven, variable patterns in *Zea mays*.

The analysis of pollution indices in the Moatize region, including Igeo, EF, CF, Cd, Er, Tu, and TRI, confirms that the area suffers from severe and widespread contamination. These indices collectively indicate a significant anthropogenic impact that far exceeds natural background levels (Sigue et al., 2025; Tomczyk et al., 2023). The Igeo and EF effectively distinguish between geogenic and anthropogenic pollution, showing that elements such as U and Co are highly enriched, thereby confirming their anthropogenic origin (Tiabou et al., 2024a, 2024b, 2024c). To connect soil geochemistry to ecological health, this study utilised the Er, which compares chemical concentrations with the environment’s specific toxicological sensitivity. While the CF quantifies the overall contamination level, the Er assesses potential environmental damage by factoring in an element’s toxic response. In this study, both As and Co pose a severe risk due to their high toxicity.

This ecological risk is further elucidated by the toxicity disparity between Moatize’s soil and sediment, which highlights distinct accumulation patterns. The terrestrial environment serves as the primary sink for pollutants, as evidenced by elevated TU and TRI values indicating greater acute stress in soils than in the aquatic compartment. In the latter, fluvial dilution likely mitigates toxicity (Zhang et al., 2016). Despite these differences in intensity, both media share a consistent risk hierarchy, identifying a common geochemical source. The dominance of As and Cr is particularly concerning; their high intrinsic toxicity poses a disproportionate ecological threat, even at lower concentrations (Raj & Maiti, 2020). By correlating soil PTE levels with the concentrations found in Zea mays and Vigna unguiculata, we go beyond mere chemical presence to demonstrate active biological transfer. This combined approach confirms that the geochemical signature of the Moatize region presents a tangible threat to the local food chain. This risk is especially high under the acidic conditions observed in the study area, which increase metal mobility and facilitate the transfer of toxic elements from the soil into staple crops (Zhang et al., 2023).

The high PLI and Cd values integrate these individual findings, confirming a severe overall pollution status. These results mirror findings in other mining-impacted regions (Afahnwie et al., 2025c, 2025d). For instance, a study in Mexico found that while individual indices showed high contamination for specific elements, broader indices such as Cd and PLI occasionally indicated moderate levels across a wider range of samples (Duarte Zaragoza & González, 2025). Similarly, elevated values across all these indices have been reported for most toxic elements in Indian coal mining areas (Chakraborty et al., 2023).

In the Moatize region, cowpea consistently accumulated higher concentrations of PTEs than *Zea mays*, highlighting the critical influence of plant genetics and species on contaminant absorption (Priya et al., 2023). These crop-specific differences in PTE accumulation are likely driven by the contrasting root morphologies and physiological mechanisms of the two species. While Zea mays may naturally exclude certain elements, such as Al at the root barrier, the symbiotic relationships and rhizosphere acidification common in legumes like Cowpea can enhance the solubility and subsequent uptake of contaminants like Cr and Ni (Hasan et al., 2025). For instance, Cr, Mo, and Ni were frequently undetectable in maize samples. Since both maize and cowpea are essential staple foods in Moatize and throughout central and southern Mozambique, their contamination presents a major public health crisis, as daily consumption exposes both adults and children to these metals. Adults generally exhibited a higher DMI, which correlates with increased carcinogenic risk, a trend consistent with recent research on age-specific contaminant vulnerability (Wang et al., 2023). The significant intake of Fe and Al reflects their widespread environmental presence and high accumulation rates in specific crops, aligning with broader studies on dietary metal exposure (Budi et al., 2024). Notably, cowpea consumption emerged as a primary exposure pathway for adults (Antoniadis et al., 2017). Furthermore, while children’s DMI may be numerically lower than that of adults, their inherent physiological sensitivity and lower body mass make them more susceptible to adverse health effects even at lower exposure levels (Bala et al., 2024).

Monitoring non-regulated elements such as Mo, S, V, and Si is essential for a comprehensive health risk assessment, as the lack of FAO/WHO regulatory limits does not equate to an absence of risk. In this study, sulphur acts as a key indicator of soil acidification, a process that increases the bioavailability of regulated PTEs such as Al, Fe, and Mn (Mensah & Amoakwah, 2024). Furthermore, V and Mo are emerging contaminants that can cause phytotoxicity and livestock disorders such as molybdenosis (Costa & Coutinho, 2022). Including these elements, along with observed exceedances of Cr and Ni, enables a more complete understanding of elemental transfer. The results emphasise *Vigna unguiculata*’s significantly greater susceptibility to bioaccumulation compared to *Zea mays*.

Health risk findings must be interpreted within the context of local dietary habits. In Moatize, maize and cowpea are staple foods, accounting for over 70% of the daily caloric intake for most households. This high frequency and duration of exposure, together with large quantities consumed, contribute to a cumulative toxicological burden (Pekmezci et al., 2025). Our results showed that although individual metal concentrations may appear moderate, the HI, which sums the risks of all PTEs, exceeds safe thresholds for both adults and children. This is particularly concerning for children, whose smaller body mass (BW) and higher metabolic rates lead to a disproportionately high intake of PTEs per kilogram of body weight (Sedghi et al., 2025). The reliance on river-irrigated floodplains for ’dry season’ farming further exposes these communities to contaminants year-round, making the dietary pathway the primary driver of the calculated risk. In Moatize, the observed Cr exceeds acceptable thresholds. According to US Epa (1989) standards and research by Yang et al. (2023), these values indicate a lifetime cancer risk above the tolerable level. Children exhibit greater susceptibility than adults to both the carcinogenic and non-carcinogenic risks posed by these toxic elements, a vulnerability similarly highlighted by Ramires et al. (2023) in Brazil.

## Conclusion

This study provides a comprehensive quantitative assessment of PTE contamination in the Moatize coal mining area. Data confirm that 100% of soil samples exceeded FAO/WHO permissible limits for Al (up to 8,112,000 mg/kg), As (up to 67.5 mg/kg), Fe (up to 152,700 mg/kg), and Mn (up to 1,221 mg/kg). Furthermore, EC in all samples exceeded the regulatory threshold of 4 μS/m, reaching a peak of 61.6 μS/m. The ecological risk is evidenced by high enrichment factors (EF > 2) for As, Co, and Ni, alongside extreme risk indices (Er > 320) for As and Co, which directly identify mining as the primary pollution source. Notably, Moatize soils exhibit significantly higher ecological stress than sediments; TU peaked at 5.94 in soils versus 4.3 in sediments, with 65% of soil samples classified as "very high toxic risk" compared to a "considerable" risk peak in sediments. Despite these differences in intensity, a consistent risk hierarchy across both media identifies As and Cr as the primary toxicity drivers. Health risk assessments revealed that 60% of food crops exceeded the 0.1 mg/kg safety limit for Cr and Ni. Furthermore, non-carcinogenic risks were confirmed by Hazard Quotients (HQ) exceeding 1.0 for Al, Mn, and Fe in adults. These data-driven findings highlight an urgent need for regular monitoring and targeted land-use interventions to safeguard the Moatize ecosystem and its residents. A comprehensive environmental management and remediation strategy is essential. Efforts should focus on reducing soil contamination, encouraging the cultivation of crop varieties with lower accumulation capacities, and educating local communities about safer dietary practices to minimise exposure. This research is highly relevant to populations residing near coal mines. By guiding risk mitigation in data-limited, rapidly urbanising areas, the study directly contributes to Sustainable Development Goal 11: Sustainable Cities and Communities.

## Supplementary Information

Below is the link to the electronic supplementary material.

ESM1 (DOC 343 KB)

## Data Availability

Data used in this study are available on request.
