# Supplementary material for: Human health risks of potentially toxic elements in the soil-crop system of a coal mining area, Moatize, Mozambique
Source: Environ Monit Assess. 2026 Apr 10;198(5):434. doi: 10.1007/s10661-026-15147-x (PMC13068753; doi:10.1007/s10661-026-15147-x)
Supplement: Supplementary file 1 — (DOC 343 KB) [file 10661_2026_15147_MOESM1_ESM.doc]

**Human Health Risk from Potentially Toxic Elements in the Soil-Crop System of a Coal Mining Area: A Case Study from Moatize, Mozambique**

Micaela Arlete Jose Chapo Cossa^1^, Hassina Mouri^1, *^, Robert B. Finkelman^2^, Kim Dowling^3^, Vicente Albino Manjate^4^

^1^ Department of Geology, Faculty of Science, University of Johannesburg, Johannesburg 2006, South Africa.

^2^ University of Texas at Dallas, Richardson, TX 75080, United States

^3^ School of Science, STEM College, RMIT University, Melbourne, VIC 3001, Australia

^4^ National Institute of Mines, Ministry of Mineral Resources and Energy, Maputo – Mozambique

*Corresponding Author’s E-mail: [hmouri@uj.ac.za](mailto:hmouri@uj.ac.za)

*Tel: +2711 559 4706

**Supplementary Information**

**Table S1** Statistical Analysis of Pollution Indices for Soils and Sediments in Moatize

| Elements | **Igeo** | | | | | | **EF** | | | | | | **CF** | | | | | |
| --- | --- | --- | --- | --- | --- | --- | --- | --- | --- | --- | --- | --- | --- | --- | --- | --- | --- | --- |
|  | **Sed** | | | **Soil** | | | **Sed** | | | **Soil** | | | **Sed** | | | **Soil** | | |
|  | Min | Max | Mean | Min | Max | Mean | Min | Max | Mean | Min | Max | Mean | Min | Max | Mean | Min | Max | Mean |
| As | 2.1 | 3.9 | 3.2 | 0.4 | 5.5 | 3.9 | -15.4 | 4.2 | -1.6 | 0.4 | 15.3 | 7.1 | 1.8 | 13.0 | 4.91 | 1.8 | 67.5 | 29.0 |
| Al | 1.1 | 1.7 | 1.3 | 0.9 | 7.8 | 1.6 | 0.4 | 0.6 | 0.6 | 0.5 | 60.3 | 4.1 | 3.3 | 4.8 | 3.7 | 2.9 | 324.5 | 16.7 |
| Ba | -0.8 | -0.3 | -0.5 | -3.0 | -0.3 | -0.9 | 0.16 | 3.6 | 1.7 | 0.1 | 0.7 | 0.2 | 0.9 | 27.9 | 12.1 | 0.2 | 27.9 | 4.1 |
| Co | 6.4 | 7.3 | 6.8 | 5.4 | 7.6 | 6.5 | 0.0 | 43.1 | 15.7 | 0.6 | 87.4 | 29.5 | 0.0 | 231.6 | 97.3 | 0.0 | 293.0 | 140.8 |
| Cr | 04 | 0.5 | 0.4 | -1.3 | 1.3 | 0.1 | 0.3 | 0.4 | 0.3 | 0.1 | 0.7 | 0.4 | 1.9 | 2.1 | 2.0 | 0.6 | 3.8 | 1.8 |
| Cu | 0.6 | 1.9 | 1.3 | -0.9 | 1.1 | 0.3 | 0.4 | 1.06 | 0.6 | 0.2 | 1.1 | 0.4 | 2.3 | 5.7 | 4.0 | 0.8 | 5.7 | 2.7 |
| Fe | 1.8 | 2.4 | 2.2 | 0.9 | 3.4 | 2.0 | 1.0 | 1.0 | 1.0 | 0.5 | 2.9 | 1.2 | 5.4 | 8.1 | 6.9 | 2.7 | 15.6 | 6.6 |
| Mn | -0.9 | -0.5 | -0.7 | -2.3 | -0.4 | -1.0 | 0.1 | 0.2 | 0.1 | 0.1 | 0. 7 | 0.2 | 0.8 | 1.0 | 0.9 | 0.3 | 1.1 | 0.8 |
| Ni | 3.1 | 3.7 | 3.5 | 1.8 | 3.7 | 2.9 | 2.1 | 3.6 | 2.7 | 0.6 | 4.9 | 2.3 | 13.3 | 19.6 | 17.2 | 5.1 | 20.1 | 13.2 |
| Pb | -0.2 | 2.1 | 0.9 | -1.9 | 2.9 | -0.1 | 0.0 | 0.2 | 0.1 | 0.1 | 2.1 | 0.4 | 0.0 | 6.2 | 2.5 | 0.0 | 11.3 | 2.2 |
| S | -1.4 | 2. 9 | 0.7 | -1.9 | 1.1 | -0.8 | 0.0 | 1.3 | 0.4 | 0.0 | 5.0 | 0.5 | 0.0 | 7.1 | 2.0 | 0.0 | 7.1 | 1.7 |
| Si | -15.9 | -12.5 | -14.2 | -19.5 | -9.9 | -14.1 | 0.0 | 20007.0 | 5001.0 | 0.0 | 0.10 | 0.01 | 0.00 | 0.40 | 0.13 | 0.0 | 0.0 | 0.0 |
| U | 16.2 | 16.8 | 16.5 | 2.6 | 2.6 | 2.6 | 18859.3 | 20541.7 | 19936.9 | 1.0 | 20339.7 | 1131.5 | 109377. 8 | 165633.3 | 138027. 8 | 8. 9 | 165633.3 | 42987. 6 |
| V | 1.83 | 2.0 | 1.9 | -1.7 | 2.8 | 1.5 | 0.7 | 0.9 | 0.8 | 0.1 | 2.0 | 0.9 | 5.4 | 5.8 | 5.5 | 0. 5 | 10.7 | 5.2 |
| Zn | 1.8 | 2.2 | 1.9 | -0.4 | 2.2 | 1.1 | 0. 7 | 1.3 | 0.9 | 0.2 | 1.5 | 0.7 | 5.2 | 6.8 | 5.7 | 1.1 | 6.8 | 4.1 |
| **Er** |  |  |  |  |  |  |  |  |  |  |  |  |  |  |  |  |  |  |
| Elements | **Sed** | | | **Soil** | | |  |  |  |  |  |  |  |  |  |  |  |  |
|  | Min | Max | Mean | Min | Max | Mean |  |  |  |  |  |  |  |  |  |  |  |  |
| As | 62.0 | 226.2 | 168.7 | 20.0 | 675.2 | 400.7 |  |  |  |  |  |  |  |  |  |  |  |  |
| Co | 0.1 | 1158 | 620.8 | 311. 7 | 1280 | 621.9 |  |  |  |  |  |  |  |  |  |  |  |  |
| Cr | 3. 9 | 4.2 | 4.0 | 1.2 | 7.6 | 3.7 |  |  |  |  |  |  |  |  |  |  |  |  |
| Cu | 11. 7 | 28.4 | 21. 7 | 3.9 | 16.2 | 9.6 |  |  |  |  |  |  |  |  |  |  |  |  |
| Fe | 5.4 | 8.1 | 6.6 | 2.7 | 15.6 | 6.8 |  |  |  |  |  |  |  |  |  |  |  |  |
| Mn | 0.8 | 1.0 | 0.9 | 0.3 | 1.1 | 0.7 |  |  |  |  |  |  |  |  |  |  |  |  |
| Ni | 0.0 | 97.8 | 70.9 | 25.1 | 100.3 | 58.1 |  |  |  |  |  |  |  |  |  |  |  |  |
| Pb | 0.0 | 31. 9 | 8.8 | 2.1 | 56.4 | 12.1 |  |  |  |  |  |  |  |  |  |  |  |  |
| Zn | 5.2 | 6.8 | 5.9 | 1.1 | 5.5 | 3.2 |  |  |  |  |  |  |  |  |  |  |  |  |

**Table S2** Pollution Indices for Sediments and Soils in Moatize

| Index | SId | **Potential Toxic Elements** | | | | | | | | | | | | | | |
| --- | --- | --- | --- | --- | --- | --- | --- | --- | --- | --- | --- | --- | --- | --- | --- | --- |
|  |  | As | Al | Ba | Co | Cr | Cu | Fe | Mn | Ni | Pb | S | Si | U | V | Zn |
| **Igeo** | Sd1 | **3.83** | 1.14 | -0.39 | **7.27** | 0.38 | 1.92 | 1.84 | -0.88 | **3.70** | -0.23 | 2.25 | -15.98 | **16.15** | 1.83 | **2.17** |
|  | Sd2 | **2.05** | 1.13 | -0.79 | **6.71** | 0.49 | 0.99 | **2.43** | -0.52 |  |  | -1.37 | -14.12 | **16.75** | 1.96 | 1.84 |
|  | Sd3 | **3.91** | 1.69 | -0.03 | **6.44** | 0.40 | 1.73 | **2.38** | -0.80 | **3.62** | 2.06 | 2.89 | -12.48 | **16.67** | 1.87 | 1.81 |
|  | Sd4 | **3.12** | 1.21 | -0.65 | **6.95** | 0.39 | 0.64 | **2.09** | -0.70 | **3.14** |  | -1.01 | -14.08 | **16.29** | 1.86 | 1.80 |
|  | S1 | **4.78** | 1.91 | -0.19 | **7.23** | -0.24 | 0.43 | **2.11** | -1.10 | **2.50** | -1.22 | -0.11 | -15.44 | **2.57** | 1.83 | **2.17** |
|  | S2 | **4.80** | 1.71 | -1.20 | **6.93** | -0.43 | 0.45 | **2.00** | -1.06 | **2.42** | -0.56 | 0.78 | -19.59 | bdl | 1.19 | 0.60 |
|  | S3 | **5.29** | 1.75 | -0.66 | **7.61** | 0.27 | 0.83 | **2.23** | -0.47 | **3.28** | 0.63 | -1.53 | -16.27 | bdl | 1.73 | 1.52 |
|  | S4 | **5.28** | 1.78 | -0.88 | **7.43** | 0.49 | 1.02 | **2.37** | -0.44 | **3.55** | 0.59 | -0.95 | -16.27 | bdl | 1.85 | 1.57 |
|  | S5 | **4.28** | 1.27 | -0.16 | **6.72** | 0.17 | 0.70 | **2.13** | -1.00 | **2.91** | 0.41 | -0.34 | -13.99 | bdl | 1.74 | 0.98 |
|  | S6 | 0.42 | 7.76 | -2.92 | **6.50** | -1.34 | -0.94 | 0.86 | -2.34 | **1.77** | -1.11 | -0.34 | -10.72 | bdl | 0.73 | -0.42 |
|  | S7 | **3.76** | 0.95 | -2.10 | **7.23** | 0.24 | -0.59 | **2.05** | -1.04 | **2.66** | 0.47 | -1.23 | -16.27 | bdl | 1.95 | 0.78 |
|  | S8 | **4.38** | 1.32 | -2.70 | **7.42** | 1.34 | -0.19 | **2.54** | -0.84 | **3.74** | -1.87 | -1.33 | -16.27 | bdl | **2.52** | 0.79 |
|  | S9 | **2.44** | 1.39 | -0.41 | **6.98** | 0.55 | 1.11 | **2.52** | -0.84 | **3.59** | 0.47 | -0.33 | -13.63 | bdl | **2.04** | 1.76 |
|  | S10 | 0.42 | 1.23 | -1.54 | **6.28** | -0.29 | 0.53 | 0.99 | -0.85 | **3.15** | -0.70 | -1.86 | -16.27 | bdl | -1.74 | 1.31 |
|  | S11 | **4.87** | 1.29 | -1.63 | **5.80** | -0.33 | -0.33 | 1.90 | -1.36 | **2.10** | -0.84 | -1.08 | -12.94 | bdl | 1.32 | 0.29 |
|  | S12 | **4.96** | 1.20 | -0.16 | **5.66** | 0.14 | 0.18 | 1.92 | -1.02 | **2.75** | -0.09 | -0.54 | -16.11 | bdl | 1.58 | 0.98 |
|  | S13 | **5.01** | 1.17 | -1.63 | **6.14** | -0.29 | 0.75 | 1.18 | -1.17 | **2.52** | 0.33 | 0.28 | -11.79 | bdl | 1.34 | 0.66 |
|  | S14 | **5.28** | 1.21 | -1.54 | **5.87** | 0.26 | 0.40 | **2.31** | -1.07 | **2.81** | -0.08 | 1.07 | -13.09 | bdl | 1.90 | 1.86 |
|  | S15 | **4.53** | 1.30 | -0.83 | **5.38** | 0.04 | 0.44 | **2.01** | -1.13 | **2.50** | 2.91 | -1.37 | -13.29 | bdl | 1.61 | 1.86 |
|  | S16 | **5.49** | 1.56 | -1.14 | **6.98** | 0.39 | 0.87 | **2.14** | -0.92 | **3.18** | 0.03 | -1.75 | -10.87 | bdl | 1.95 | 0.87 |
|  | S17 | **5.14** | 1.14 | -0.82 | **5.70** | 0.87 | 0.27 | **3.38** | -0.41 | **3.46** | 0.21 | -1.98 | -9.89 | bdl | **2.84** | 1.05 |
|  | S18 | **5.33** | 1.52 | -1.07 | **5.60** | 0.10 | -0.02 | 1.52 | -1.69 | **2.66** | -1.87 | -0.93 | -11.45 | bdl | 1.36 | 0.77 |
| **EF** | Sd1 | -15.44 | 0.62 | 0.21 | **43.07** | 0.36 | 1.06 | 1.00 | 0.15 | **3.64** | 0.24 | 1.32 | 0.00 | 20339.70 | 0.99 | 1.26 |
|  | Sd2 | **4.23** | 0.41 | 0.11 | **19.54** | 0.26 | 0.37 | 1.00 | 0.13 |  | 0.00 | 0.07 | 0.00 | 20541.72 | 0.73 | 0.67 |
|  | Sd3 | **2.98** | 0.62 | **3.57** | 0.00 | 0.25 | 0.64 | 1.00 | 0.11 | **2.36** | 0.05 | 0.05 | **20007.2** | 20007.18 | 0.70 | 0.67 |
|  | Sd4 | **1.80** | 0.54 | **2.86** | 0.00 | 0.31 | 0.37 | 1.00 | 0.14 | **2.08** | 0.00 | 0.00 | 0.02 | 18859.34 | 0.85 | 0.82 |
|  | S1 | **3.96** | 0.62 | 0.21 | **43.07** | 0.36 | 1.06 | 1.00 | 0.15 | **3.64** | 0.24 | 1.32 | 0.00 | 20339.70 | 0.99 | 1.26 |
|  | S2 | **7.77** | 0.92 | 0.12 | **33.97** | 0.21 | 0.38 | 1.11 | 0.13 | 1.49 | 0.19 | 0.48 | 0.00 | bdl | 0.64 | 0.42 |
|  | S3 | **10.90** | 0.94 | 0.18 | **54.49** | 0.34 | 0.50 | 1.31 | 0.20 | **2.72** | 0.43 | 0.10 | 0.00 | bdl | 0.92 | 0.80 |
|  | S4 | 0.71 | 0.97 | 0.69 | 0.62 | 0.62 | 0.77 | 0.85 | 0.67 | 0.55 | 0.44 | **4.96** | 0.10 | bdl | 0.69 | 0.53 |
|  | S5 | **15.29** | 0.96 | 0.26 | **87.40** | 0.55 | 0.64 | 1.53 | 0.30 | **4.97** | 0.98 | 0.02 | 0.00 | bdl | 1.34 | 1.51 |
|  | S6 | 0.37 | **60.34** | 0.04 | **25.29** | 0.11 | 0.15 | 0.50 | 0.05 | 0.95 | 0.13 | 0.22 | 0.00 | bdl | 0.46 | 0.21 |
|  | S7 | **3.78** | 0.54 | 0.07 | **41.90** | 0.33 | 0.19 | 1.16 | 0.14 | 1.77 | 0.39 | 0.12 | 0.00 | bdl | 1.08 | 0.48 |
|  | S8 | **5.80** | 0.70 | 0.04 | **47.61** | 0.71 | 0.24 | 1.62 | 0.16 | **3.73** | 0.08 | 0.11 | 0.00 | bdl | 1.60 | 0.48 |
|  | S9 | 1.51 | 0.73 | 0.21 | **35.21** | 0.41 | 0.60 | 1.61 | 0.16 | **3.36** | 0.39 | 0.22 | 0.00 | bdl | 1.14 | 0.94 |
|  | S10 | 0.37 | 0.65 | 0.10 | **21.63** | 0.23 | 0.40 | 0.55 | 0.15 | **2.48** | 0.17 | 0.08 | 0.00 | bdl | 0.08 | 0.69 |
|  | S11 | 8.17 | 0.68 | 0.09 | **15.56** | 0.22 | 0.22 | 1.04 | 0.11 | 1.20 | 0.16 | 0.13 | 0.00 | bdl | 0.70 | 0.34 |
|  | S12 | **8.70** | 0.64 | 0.25 | **14.13** | 0.31 | 0.32 | 1.06 | 0.14 | 1.88 | 0.26 | 0.19 | 0.00 | bdl | 0.83 | 0.55 |
|  | S13 | **8.98** | 0.63 | 0.09 | **19.65** | 0.23 | 0.47 | 0.63 | 0.12 | 1.60 | 0.35 | 0.34 | 0.00 | bdl | 0.70 | 0.44 |
|  | S14 | **10.82** | 0.65 | 0.10 | **16.36** | 0.33 | 0.37 | 1.38 | 0.13 | 1.95 | 0.26 | 0.59 | 0.00 | bdl | 1.04 | 1.02 |
|  | S15 | **6.45** | 0.69 | 0.16 | **11.59** | 0.29 | 0.38 | 1.13 | 0.13 | 1.58 | **2.10** | 0.11 | 0.00 | bdl | 0.85 | 1.02 |
|  | S16 | **12.56** | 0.82 | 0.13 | **35.21** | 0.37 | 0.51 | 1.23 | 0.15 | **2.52** | 0.29 | 0.08 | 0.00 | bdl | 1.08 | 0.51 |
|  | S17 | **9.87** | 0.61 | 0.16 | **14.50** | 0.51 | 0.34 | **2.90** | 0.21 | **3.07** | 0.32 | 0.07 | 0.00 | bdl | **2.00** | 0.58 |
|  | S18 | **11.21** | 0.80 | 0.13 | **13.51** | 0.30 | 0.28 | 0.80 | 0.09 | 1.76 | 0.08 | 0.15 | 0.00 | bdl | 0.72 | 0.48 |
| **CF** | Sd1 | **2.57** | **3.31** | **1.14** | **231.60** | **1.95** | **5.68** | **5.38** | 0.81 | **19.56** | **1.28** | **7.13** | 0.00 | **109377.78** | **5.35** | **6.75** |
|  | Sd2 | **1.83** | **3.29** | 0.87 | **157.57** | **2.11** | **2.99** | **8.06** | **1.04** |  |  | 0.58 | 0.00 | **165633.33** | **5.85** | **5.37** |
|  | Sd3 | **2.17** | **4.82** | **27.94** | 0.01 | **1.98** | **4.98** | **7.83** | 0.86 | **18.50** | **6.24** | 0.38 | 0.40 | **156688.89** | **5.50** | **5.25** |
|  | Sd4 | **13.04** | **3.47** | **18.23** | 0.02 | **1.97** | **2.33** | **6.38** | 0.92 | **13.25** | 0.00 | 0.03 | **0.13** | **120411.11** | **5.45** | **5.24** |
|  | S1 | **21.31** | **3.31** | **1.14** | **231.60** | **1.95** | **5.68** | **5.38** | 0.81 | **19.56** | **1.28** | **7.13** | 0.00 | **109377.78** | **5.35** | **6.75** |
|  | S2 | **41.78** | **4.92** | 0.65 | **182.67** | **1.11** | **2.05** | **5.99** | 0.72 | **8.01** | **1.02** | **2.58** | 0.00 | bdl | **3.42** | **2.28** |
|  | S3 | **58.62** | **5.05** | 0.95 | **293.00** | **1.81** | **2.66** | **7.02** | **1.08** | **14.62** | **2.32** | 0.52 | 0.00 | bdl | **4.97** | **4.30** |
|  | S4 | **58.28** | **5.16** | 0.81 | **258.67** | **2.10** | **3.03** | **7.73** | **1.11** | **17.53** | **2.26** | 0.78 | 0.00 | bdl | **5.40** | **4.46** |
|  | S5 | **29.24** | **3.61** | **1.34** | **158.00** | **1.69** | **2.44** | **6.56** | 0.75 | **11.26** | **1.99** | **1.19** | 0.00 | bdl | **5.00** | **2.97** |
|  | S6 | **2.00** | **324.48** | 0.20 | **136.00** | 0.59 | 0.78 | **2.71** | 0.30 | **5.11** | 0.70 | **1.19** | 0.00 | bdl | **2.49** | **1.12** |
|  | S7 | **20.32** | **2.90** | 0.35 | **225.33** | **1.78** | 1.00 | **6.22** | 0.73 | **9.50** | **2.08** | 0.64 | 0.00 | bdl | **5.80** | **2.57** |
|  | S8 | **31.20** | **3.74** | 0.23 | **256.00** | **3.80** | **1.31** | **8.69** | 0.84 | **20.06** | 0.41 | 0.60 | 0.00 | bdl | **8.60** | **2.59** |
|  | S9 | **8.14** | **3.94** | **1.13** | **189.33** | **2.19** | **3.23** | **8.63** | 0.84 | **18.05** | **2.08** | 1.20 | 0.00 | bdl | **6.15** | **5.08** |
|  | S10 | **2.00** | **3.52** | 0.52 | **116.33** | **1.23** | **2.16** | **2.98** | 0.83 | **13.33** | 0.92 | 0.41 | 0.00 | bdl | 0.45 | **3.71** |
|  | S11 | **43.96** | **3.68** | 0.48 | **83.67** | **1.19** | **1.20** | **5.58** | 0.59 | **6.44** | 0.84 | 0.71 | 0.00 | bdl | 3.74 | **1.84** |
|  | S12 | **46.77** | **3.44** | **1.34** | **76.00** | **1.66** | **1.70** | **5.67** | 0.74 | **10.12** | **1.41** | **1.03** | 0.00 | bdl | 4.49 | **2.97** |
|  | S13 | **48.27** | **3.38** | 0.48 | **105.67** | **1.22** | **2.52** | **3.39** | 0.67 | **8.58** | **1.88** | **1.82** | 0.00 | bdl | 3.79 | **2.37** |
|  | S14 | **58.21** | **3.47** | 0.52 | **88.00** | **1.80** | **1.98** | **7.44** | 0.72 | **10.50** | **1.42** | **3.15** | 0.00 | bdl | 5.60 | **5.46** |
|  | S15 | **34.68** | **3.70** | 0.84 | **62.33** | **1.54** | **2.04** | **6.06** | 0.69 | **8.48** | **11.29** | 0.58 | 0.00 | bdl | 4.57 | **5.46** |
|  | S16 | **67.52** | **4.43** | 0.68 | **189.33** | **1.96** | **2.74** | **6.59** | 0.79 | **13.56** | **1.53** | 0.45 | 0.00 | bdl | 5.80 | **2.75** |
|  | S17 | **53.07** | **3.30** | 0.85 | **78.00** | **2.74** | **1.80** | **15.58** | **1.13** | **16.54** | **1.74** | 0.38 | 0.00 | bdl | 10.75 | **3.10** |
|  | S18 | **60.30** | **4.29** | 0.72 | **72.67** | **1.61** | **1.48** | **4.30** | 0.46 | **9.49** | 0.41 | 0.79 | 0.00 | bdl | 3.86 | **2.56** |
| **Er** | Sd1 | **213.10** | bdl | bdl | **1158.00** | 3.89 | 28.41 | 5.38 | 0.81 | **97.78** | 6.39 | bdl | bdl | bdl | bdl | 6.75 |
|  | Sd2 | **62.00** | bdl | bdl | **787.83** | 4.21 | 14.93 | 8.06 | 1.04 | 0.00 | 0.00 | bdl | bdl | bdl | bdl | 5.37 |
|  | Sd3 | **226.20** | bdl | bdl | 0.06 | 3.95 | 24.92 | 7.83 | 0.86 | **92.50** | 31.19 | bdl | bdl | bdl | bdl | 5.25 |
|  | Sd4 | **130.40** | bdl | bdl | 0.08 | 3.93 | 11.67 | 6.38 | 0.92 | **66.25** | 0.00 | bdl | bdl | bdl | bdl | 5.24 |
|  | S1 | **213.10** | bdl | bdl | **1158.00** | 3.89 | 28.41 | 5.38 | 0.81 | **97.78** | 6.39 | bdl | bdl | bdl | bdl | 6.75 |
|  | S2 | **417.80** | bdl | bdl | **913.33** | 2.22 | 10.27 | 5.99 | 0.72 | 40.03 | 5.10 | bdl | bdl | bdl | bdl | 2.28 |
|  | S3 | **586.20** | bdl | bdl | **1465.00** | 3.61 | 13.31 | 7.02 | 1.08 | **73.10** | 11.60 | bdl | bdl | bdl | bdl | 4.30 |
|  | S4 | **582.80** | bdl | bdl | **1293.33** | 4.20 | 15.16 | 7.73 | 1.11 | **87.65** | 11.31 | bdl | bdl | bdl | bdl | 4.46 |
|  | S5 | **292.40** | bdl | bdl | **790.00** | 3.38 | 12.19 | 6.56 | 0.75 | **56.28** | 9.97 | bdl | bdl | bdl | bdl | 2.97 |
|  | S6 | 20.00 | bdl | bdl | **680.00** | 1.18 | 3.91 | 2.71 | 0.30 | 25.55 | 3.49 | bdl | bdl | bdl | bdl | 1.12 |
|  | S7 | **203.20** | bdl | bdl | **1126.67** | 3.55 | 4.98 | 6.22 | 0.73 | **47.48** | 10.39 | bdl | bdl | bdl | bdl | 2.57 |
|  | S8 | **312.00** | bdl | bdl | **1280.00** | 7.60 | 6.57 | 8.69 | 0.84 | **100.28** | 2.05 | bdl | bdl | bdl | bdl | 2.59 |
|  | S9 | **81.40** | bdl | bdl | **946.67** | 4.38 | 16.17 | 8.63 | 0.84 | **90.23** | 10.39 | bdl | bdl | bdl | bdl | 5.08 |
|  | S10 | 20.00 | bdl | bdl | **581.67** | 2.46 | 10.81 | 2.98 | 0.83 | **66.65** | 4.62 | bdl | bdl | bdl | bdl | 3.71 |
|  | S11 | **439.60** | bdl | bdl | **418.33** | 2.38 | 5.98 | 5.58 | 0.59 | 32.20 | 4.19 | bdl | bdl | bdl | bdl | 1.84 |
|  | S12 | **467.70** | bdl | bdl | **380.00** | 3.31 | 8.48 | 5.67 | 0.74 | **50.60** | 7.06 |  |  |  |  | 2.97 |
|  | S13 | **482.70** | bdl | bdl | **528.33** | 2.45 | 12.58 | 3.39 | 0.67 | **42.90** | 9.42 | bdl | bdl | bdl | bdl | 2.37 |
|  | S14 | **582.10** | bdl | bdl | **440.00** | 3.59 | 9.90 | 7.44 | 0.72 | **52.50** | 7.09 | bdl | bdl | bdl | bdl | 5.46 |
|  | S15 | **346.80** | bdl | bdl | **311.67** | 3.09 | 10.20 | 6.06 | 0.69 | **42.38** | **56.44** | bdl | bdl | bdl | bdl | 5.46 |
|  | S16 | **675.20** | bdl | bdl | **946.67** | 3.93 | 13.70 | 6.59 | 0.79 | **67.80** | 7.67 | bdl | bdl | bdl | bdl | 2.75 |
|  | S17 | **530.70** | bdl | bdl | **390.00** | 5.47 | 9.02 | 15.58 | 1.13 | **82.68** | 8.70 | bdl | bdl | bdl | bdl | 3.10 |
|  | S18 | **603.00** | bdl | bdl | **363.33** | 3.21 | 7.40 | 4.30 | 0.46 | **47.43** | 2.06 |  |  |  |  | 2.56 |
| **CD** |  | **748.84** | **405.22** | **17.66** | **3191.80** | **39.96** | **47.80** | **144.20** | **17.42** | **273.00** | **45.10** | **33.24** | 0.54 | **552111.11** | **242.85** | **84.92** |
| **PLI** |  | **24.32** | **4.34** | 0.72 | **51.66** | **1.67** | **1.94** | **5.65** | 0.77 | **9.73** | **1.45** | 0.85 | 0.37 | **7.81** | **9.07** | **3.33** |

bdl- below detection limit

**Table S3** Daily Metal Intake (DMI) in the Population of Moatize

| Adults | | | | | | | | | | | |
| --- | --- | --- | --- | --- | --- | --- | --- | --- | --- | --- | --- |
|  | Al | Cr | Cu | Fe | Mn | Mo | Ni | S | V | Zn | Si |
| Zm1 | 838.4 | - | 0.0009 | 1356.6 | 0.0133 | 0.00034 | - | 0.4855 | 0.0003 | 0.0067 | 0.0819 |
| Zm2 | 533.2 | - | - | 602.1 | 0.0093 | - | - | 0.4019 | - | 0.0053 | 0.0679 |
| Zm3 | 874 | - | 0.0011 | 361.9 | 0.0211 | - | - | 0.5651 | 0.0004 | 0.0113 | 0.41 |
| Zm4 | 874 | - | - | 223.3 | 0.018 | - | - | 0.3713 | 0.0002 | 0.0031 | 0.0249 |
| Zm5 | 816.9 | - | 0.0009 | 825.8 | 0.0124 | - | - | 0.4641 | 0.0004 | 0.0146 | 0.0302 |
| Zm6 | 1101.6 | - | - | 1693.2 | 0.0119 | - | - | 0.2978 | 0.0004 | 0.0032 | 0.1295 |
| Zm7 | 748.7 | 0.0011 | 0.0021 | 1519.8 | 0.0181 | - | 0.00056 | 0.4712 | 0.0004 | 0.0136 | 0.0619 |
| Zm8 | 843.0 | 0.0012 | 0.0018 | 2182.8 | 0.0148 | 0.0002 | 0.00057 | 0.3968 | 0.0004 | 0.0101 | 0.102 |
| Zm9 | 327.1 | 0.0014 | 0.0021 | 1173.0 | 0.0138 | 0.0002 | 0.00072 | 0.4661 | 0.0003 | 0.0061 | 0.0681 |
| Zm10 | 354.5 | 0.0010 | 0.0018 | 913.1 | 0.007 | 0.0002 | 0.00048 | 0.4121 | - | 0.0069 | 0.0691 |
| C1 | 718.5 | 0.0042 | 0.0013 | 1815.6 | 0.0247 | - | 0.00056 | 0.5610 | 0.0006 | 0.0038 | 0.0692 |
| C2 | 1275 | 0.0026 | 0.0012 | 2315.4 | 0.0166 | 0.0007 | 0.0012 | 0.4468 | 0.0007 | 0.0056 | 0.1408 |
| C3 | 1387.2 | 0.0016 | 0.0012 | 3406.8 | 0.0305 | - | 0.0007 | 0.4784 | 0.0005 | 0.0077 | 0.0971 |
| C4 | 1815.6 | 0.0015 | 0.0014 | 3406.8 | 0.0294 | - | 0.00067 | 0.7446 | 0.0008 | 0.0149 | 0.1112 |
| C5 | 740.5 | 0.0009 | 0.0012 | 1581.0 | 0.0294 | - | 0.00053 | 0.5875 | 0.0008 | 0.0068 | 0.0639 |
| Mean | 883.2 | 0.0017 | 0.0014 | 1558.5 | 0.0180 | 0.0003 | 0.00067 | 0.4767 | 0.0005 | 0.008 | 0.1018 |
| **Children** | | | | | | | | | | | |
|  | Al | Cr | Cu | Fe | Mn | Mo | Ni | S | V | Zn | Si |
| Zm1 | 123.4 | - | 0.0001 | 199.7 | 0.0020 | 0.0001 | - | 0.0715 | 0.0000 | 0.0017 | 0.0604 |
| Zm2 | 78.5 | - | - | 88.6 | 0.0014 | - | - | 0.0592 | - | 0.0005 | 0.0037 |
| Zm3 | 128.7 | - | 0.0002 | 53.3 | 0.0031 | - | - | 0.0832 | 0.0001 | 0.0021 | 0.0044 |
| Zm4 | 128. 8 | - | - | 32.8 | 0.0027 | - | - | 0.0547 | 0.0000 | 0.0005 | 0.0191 |
| Zm5 | 120.3 | - | 0.0001 | 121.6 | 0.0018 | - | - | 0.0683 | 0.0001 | 0.0020 | 0.0091 |
| Zm6 | 162.2 | - | - | 249.3 | 0.0018 | - | - | 0.0438 | 0.0001 | 0.0015 | 0.0150 |
| Zm7 | 110.2 | 0.0002 | 0.0003 | 223.6 | 0.0027 | - | 0.0001 | 0.0694 | 0.0001 | 0.0009 | 0.0100 |
| Zm8 | 124.1 | 0.0002 | 0.0003 | 321.4 | 0.0022 | 0.0000 | 0.0001 | 0.0584 | 0.0001 | 0.0010 | 0.0102 |
| Zm9 | 48.2 | 0.0002 | 0.0003 | 172.7 | 0.0020 | 0.0000 | 0.0001 | 0.0686 | 0.0000 | 0.0009 | 0.0100 |
| Zm10 | 52.2 | 0.0001 | 0.0003 | 134.4 | 0.0010 | 0.0000 | 0.0001 | 0.0607 | - | 0.0010 | 0.0102 |
| C1 | 105. 8 | 0.0006 | 0.0002 | 267.3 | 0.0036 | - | 0.0001 | 0.0826 | 0.0001 | 0.0006 | 0.0102 |
| C2 | 187.7 | 0.0004 | 0.0002 | 340.9 | 0.0025 | 0.0001 | 0.0002 | 0.0658 | 0.0001 | 0.0008 | 0.0207 |
| C3 | 204.2 | 0.0002 | 0.0002 | 501.6 | 0.0045 | - | 0.0001 | 0.0704 | 0.0001 | 0.0011 | 0.0143 |
| C4 | 267.3 | 0.0002 | 0.0002 | 5017 | 0.0043 | - | 0.0001 | 0.1096 | 0.0001 | 0.0022 | 0.0164 |
| C5 | 109.0 | 0.0001 | 0.0002 | 232.7 | 0.0043 | - | 0.0001 | 0.0865 | 0.0001 | 0.0010 | 0.0094 |
| **Mean** | 130.0 | 0.0003 | 0.0002 | 229.4 | 0.0027 | 0.0001 | 0.0001 | 0.0702 | 0.0001 | 0.0012 | 0.0149 |

Zm- Zea mays; C- Cowpea

**Table S4** Hazard Risk Quotient for the Community of Moatize

| Adults | | | | | | | |
| --- | --- | --- | --- | --- | --- | --- | --- |
|  | Al | Cr | Cu | Fe | Mn | Ni | Zn |
| Zm1 | 838.4 | - | 0.0232 | 1938.0 | 0.9486 | - | 0.0223 |
| Zm2 | 533.2 | - | - | 860.2 | 0.6645 | - | 0.0176 |
| Zm3 | 874.0 | - | 0.0283 | 517.0 | 1.5089 | - | 0.0375 |
| Zm4 | 874.0 | - | - | 319.0 | 1.2867 | - | 0.0104 |
| Zm5 | 816.9 | - | 0.0224 | 1179.7 | 0.8852 | - | 0.0486 |
| Zm6 | 1101.6 | - | - | 2418.9 | 0.8524 | - | 0.0108 |
| Zm7 | 748.7 | - | 0.0515 | 2171.1 | 1.2896 | 0.0278 | 0.0453 |
| Zm8 | 843.0 | 0.0007 | 0.0456 | 3118.3 | 1.0564 | 0.0285 | 0.0335 |
| Zm9 | 327.1 | 0.0008 | 0.0528 | 1675.7 | 0.9865 | 0.0361 | 0.0204 |
| Zm10 | 354.5 | 0.0009 | 0.0446 | 65221.7 | 0.5020 | 0.0241 | 0.0232 |
| C1 | 718.5 | 0.0028 | 0.0324 | 2593.7 | 1.7631 | 0.0282 | 0.0125 |
| C2 | 1275.0 | 0.0017 | 0.0296 | 3307.7 | 1.1890 | 0.0602 | 0.0186 |
| C3 | 1387.2 | 0.0010 | 0.0311 | 4866.9 | 2.1770 | 0.0348 | 0.0256 |
| C4 | 1815.6 | 0.0010 | 0.0342 | 4866.9 | 2.1005 | 0.0335 | 0.0497 |
| C5 | 740.5 | 0.0006 | 0.0291 | 2258.6 | 2.0990 | 0.0263 | 0.0226 |
| **Mean** | 883.2 | 0.0012 | 0.0354 | 6487.5 | 1.2873 | 0.0333 | 0.0266 |
|  |  |  |  |  |  |  |  |
| **Children** | | | | | | | |
|  | Al | Cr | Cu | Fe | Mn | Ni | Zn |
| Zm1 | 123.4 | - | 0.0034 | 285.3 | 0.1397 | - | 0.0055 |
| Zm2 | 78.5 | - | - | 126.6 | 0.0978 | - | 0.0015 |
| Zm3 | 128.7 | - | 0.0042 | 76.1 | 0.2221 | - | 0.0072 |
| Zm4 | 128.7 | - | - | 47.0 | 0.1894 | - | 0.0016 |
| Zm5 | 120.3 | - | 0.0033 | 173.7 | 0.1303 | - | 0.0067 |
| Zm6 | 162.2 | - | - | 356.1 | 0.1255 | - | 0.0049 |
| Zm7 | 110.2 | 0.0001 | 0.0076 | 319.6 | 0.1899 | 0.0041 | 0.0030 |
| Zm8 | 124.1 | 0.0001 | 0.0067 | 459.1 | 0.1555 | 0.0042 | 0.0034 |
| Zm9 | 48.2 | 0.0001 | 0.0078 | 246.7 | 0.1452 | 0.0053 | 0.0030 |
| Zm10 | 52.2 | 0.0001 | 0.0066 | 192.0 | 0.2847 | 0.0035 | 0.0034 |
| C1 | 105.8 | 0.0004 | 0.0048 | 381.9 | 0.2596 | 0.0042 | 0.0018 |
| C2 | 187.7 | 0.0003 | 0.0044 | 487.0 | 0.1751 | 0.0089 | 0.0027 |
| C3 | 204.2 | 0.0002 | 0.0046 | 716.5 | 0.3205 | 0.0051 | 0.0038 |
| C4 | 267.3 | 0.0002 | 0.0050 | 716.5 | 0.3092 | 0.0049 | 0.0073 |
| C5 | 109.0 | 0.0001 | 0.0043 | 332.5 | 0.3090 | 0.0039 | 0.0033 |
| **Mean** | 130.0 | 0.0002 | 0.0052 | 327.8 | 0.2036 | 0.0049 | 0.0039 |

Zm- Zea mays; C- Cowpea

**Table S5** Cancer Risk from Staple Food Consumption in Moatize

| Adults | | | | Children | | | |
| --- | --- | --- | --- | --- | --- | --- | --- |
|  | Cr | Ni | Cu |  | Cr | Ni | Cu |
| Zm1 | _ | _ | 1.58E-03 |  | _ | _ | 2.32E-04 |
| Zm2 | _ | _ | 0.00E+00 |  | _ | _ | 0.00E+00 |
| Zm3 | _ | _ | 1.92E-03 |  | _ | _ | 2.83E-04 |
| Zm4 | _ | _ | 0.00E+00 |  | _ | _ | 0.00E+00 |
| Zm5 | _ | _ | 1.53E-03 |  | _ | _ | 2.25E-04 |
| Zm6 | _ | _ | 0.00E+00 |  | _ | _ | 0.00E+00 |
| Zm7 | _ | 4.68E-07 | 3.50E-03 |  | 7.96E-05 | 6.89E-08 | 5.16E-04 |
| Z8 | 5.87E-04 | 4.79E-07 | 3.10E-03 |  | 8.63E-05 | 7.05E-08 | 4.57E-04 |
| Zm9 | 6.83E-04 | 1.15E-06 | 3.59E-03 |  | 1.01E-04 | 8.93E-08 | 5.28E-04 |
| Zm10 | 5.07E-04 | 8.53E-07 | 3.03E-03 |  | 7.47E-05 | 5.95E-08 | 4.47E-04 |
| C1 | 2.12E-03 | 3.56E-06 | 2.20E-03 |  | 3.12E-04 | 6.98E-08 | 3.24E-04 |
| C2 | 1.31E-03 | 2.20E-06 | 2.01E-03 |  | 1.93E-04 | 1.49E-07 | 2.96E-04 |
| C3 | 7.80E-04 | 1.31E-06 | 2.12E-03 |  | 1.15E-04 | 8.60E-08 | 3.11E-04 |
| C4 | 7.65E-04 | 1.29E-06 | 2.32E-03 |  | 1.13E-04 | 8.29E-08 | 3.42E-04 |
| C5 | 4.68E-04 | 7.86E-07 | 1.98E-03 |  | 6.89E-05 | 6.50E-08 | 2.91E-04 |
| Mean | 9.03E-04 | 1.34E-06 | 1.93E-03 |  | 1.27E-04 | 8.23E-08 | 2.84E-04 |

Zm- Zea mays; C- Cowpea
